# Computational Modelling of the Impact of Evaporation on *In-Vitro* Dermal Absorption

**DOI:** 10.1007/s11095-024-03779-y

**Published:** 2024-10-07

**Authors:** Benjamin N. Deacon, Samadhi Silva, Guoping Lian, Marina Evans, Tao Chen

**Affiliations:** 1 School of Chemistry and Chemical Engineering, University of Surrey, Guildford, GU2 7XH U.K.; 2 Unilever R&D Colworth, Unilever, Sharnbrook MK44 1LQ U.K.; 3 Center for Computational Toxicology and Exposure, US EPA, ORD, RTP, NC USA

**Keywords:** Dermal Absorption, Evaporation, In silico modelling, Pharmaceuticals

## Abstract

**Purpose:**

Volatiles are common in personal care products and dermatological drugs. Determining the impact of evaporation of volatiles on skin permeation is crucial to evaluate and understand their delivery, bioavailability, efficacy and safety. We aim to develop an in-silico model to simulate the impact of evaporation on the dermal absorption of volatiles.

**Method:**

The evaporation of volatile permeants was modelled using vapour pressure as the main factor. This model considers evaporation as a passive diffusion process driven by the concentration gradient between the air-vehicle interface and the ambient environment. The evaporation model was then integrated with a previously published physiologically based pharmacokinetic (PBPK) model of skin permeation and compared with published *in vitro* permeation test data from the Cosmetics Europe ADME Task Force.

**Results:**

The evaporation-PBPK model shows improved predictions when evaporation is considered. In particular, good agreement has been obtained for the distributions in the evaporative loss, and the overall percutaneous absorption. The model is further compared with published in-silico models from the Cosmetics Europe ADME Task Force where favourable results are achieved.

**Conclusion:**

The evaporation of volatile permeants under finite dose *in vitro* permeation test conditions has been successfully predicted using a mechanistic model with the intrinsic volatility parameter vapour pressure. Integrating evaporation in PBPK modelling significantly improved the prediction of dermal delivery.

**Supplementary Information:**

The online version contains supplementary material available at 10.1007/s11095-024-03779-y.

## Introduction

Skin exposure to volatile chemicals occurs regularly from the application of skincare and dermatological products or unintended environmental pollutants [1]. Understanding dermal absorption and the impact of evaporation is paramount for effective delivery of dermatological drugs and skincare products. Among the array of methods for evaluating dermal absorption, *in-vitro* permeation tests (IVPT) are well-established tools [2, 3], traditionally designed to examine 'infinite dose' conditions where ingredient depletion from the vehicle is negligible and, in many cases, the vehicle is occluded to prevent evaporation from affecting dermal absorption. Experimental emphasis has shifted towards studying finite dose and unoccluded conditions in IVPT studies, recognising the potential impact of evaporation on dermal absorption. Unoccluded finite dosing studies also apply to environmental or occupational exposures, where the concentration may change due to removal by washing or additional/repeated exposure [4].

In-silico, physiologically based pharmacokinetic (PBPK) models are supporting tools for more informed, faster, and cost-effective dermal absorption assessment [5]. While in-silico modelling of dermal absorption has progressed significantly in the past two decades [6], the incorporation of evaporation is still limited, especially when investigating the impact of volatile permeants on IVPT results.

Several models of evaporation and dermal penetration of volatile permeants have been proposed by Kasting, Frasch and their co-workers [7–11]. In these models, evaporation is described by a mass transfer equation with the evaporation rate represented by a mass transfer coefficient. The mass transfer coefficient is empirically related to the ingredients’ vapour pressure, molecular weight, and wind speed in the environment. Dermal absorption models describe convective mass transfer coefficients under different conditions: free, intermediate, and forced convection [12], free convective conditions assume low air velocities (< 0.2 m/s) [4] [13]. However, achieving satisfactory evaporation predictions often requires further fitting of key parameters in the mass transfer rate equation due to uncertainties in the experimental conditions. Wind speed on the skin surface has a large impact on evaporation [13] but can be difficult to measure accurately. For example, Kasting *et al.,* 2008 needed to fit the air flow rate to obtain a satisfactory result for the evaporation and skin absorption of N,N-Diethyl-m-Toluamide (DEET) [14].

Some notable improvements to the modelling framework reviewed above include Amarah *et al.,* 2018 [15] use of simple physiological parameters to allow for better physical interpretation of the rate constants, and limiting the number of parameters used and the inclusion of solvent evaporation by Arce *et al*., 2019 [16]. Furthermore, Tonnis *et al*., 2022 [17] calculated evaporation with consideration of the impact of solvent dry down, vehicle pH and slowly reversible keratin binding on skin penetration.

Previous research appears to have focused on wind speed as the main factor affecting the evaporation rate [18], and requires empirical fitting of the evaporative mass transfer coefficient. The environmental wind speed typically measured does not necessarily correlate well with that on the skin surface. This correlation can be further complicated by the geometry of the donor chamber in IVPT experiments – as the donor chamber is typically a tall cylinder relative to its cross-sectional area, environmental wind speed may have a relatively small effect. Against this background, the present work investigates an alternative approach to modelling evaporation. Instead of relying on the empirical mass transfer rate, the evaporation of volatile permeants is modelled by the mechanistic diffusion equation driven by the permeant’s vapour pressure at the liquid–air interface. The evaporation model is then integrated with our previously reported PBPK dermal absorption model [18–25]. We applied this integrated model to simulate the published IVPT data under finite dose, unoccluded conditions commissioned by the Cosmetics Europe task force [26], who assessed six in-silico models (TCAT, SimCyp, DSkin, CDC, Surrey model – CosEU and DermWin) with three modelling evaporation (CDC, Surrey model – CosEU and DSkin). We show that our diffusion-based approach is in good agreement with experimental mass balance data and significantly improves the prediction of dermal absorption compared with our earlier version of the model, Surrey model – CosEU, evaluated by the Cosmetics Europe [25]. The impact of evaporation of volatile permeant on dermal absorption has been quantified.

## Methods

Throughout the Methods and this paper two models are used by the authors:

PBPK – Physiologically based pharmacokinetic model, existing dermal model without evaporation [18–25].

PBPK-E – Physiologically based pharmacokinetic model with Evaporation, dermal model with a proposed evaporation module.

### Overview of the PBPK Model

To integrate evaporation into a dermal model, the latest modelling framework developed in our group has been used [18–25]. The first version of the model was published by Chen *et al*., 2008 [19] and has undergone improvements since [18–25]. The technical details of the model are described in Chen *et al*., 2016 [23].

In brief, the PBPK model solves the partial differential equations describing the conservation of mass during the dermal absorption process. Mass transfer from the vehicle into the skin is governed by diffusion, and partition coefficients are introduced to account for the thermodynamic properties across media. The diffusion and partition coefficients of permeants in different skin tissues can be provided by the user if known, or more often are calculated based on established quantitative structure–property relationships (QSPR), as detailed in Chen *et al*., 2016 [23]. For instance, the stratum corneum is described by a two-dimensional brick-and-mortar structure, with the diffusion and partition coefficients in the lipid and corneocyte regions calculated using the QSPR equations reported by Wang *et al.,* 2010 [27]. The viable epidermis and dermis are assumed to be of a single phase, i.e. having the same diffusion and partition coefficients as those calculated via the QSPRs from Kasting et al*., 2006* [7] (or provided by the user). This simplification of the viable epidermis and dermis is deemed reasonable because the stratum corneum (SC) is the primary barrier. However, this may lead to less accurate prediction of tissue-specific concentrations.

This PBPK model was further extended to include evaporation, and by invitation was submitted to the Cosmetics Europe’s dermal in-silico model evaluation exercise [18] (referred to as Surrey model – CosEU in the present paper). The evaporation rate was modelled using the empirical mass transfer coefficient requiring wind speed, as reviewed above. This empirical equation was originally published by the US Environmental Protection Agency (EPA) for the evaporation of liquid spills [28] in the context of laboratory accidents; however, Gajjar *et al.,* 2013 [29] considered it to be applicable to dermal scenarios if appropriate input values are used. In particular, the evaporative mass transfer coefficient was calculated as follows:

1 $${K}_{evap}=\frac{1.756{e}^{-5} P {MW}^\frac{2}{3} {u}^{0.78}}{R T}$$

where ${K}_{evap}$ is the evaporative mass transfer coefficient (m s^−1^), P is vapour pressure (Pa), MW is molecular weight (Da), $u$ is the wind speed (m s^−1^), R is the Gas constant (J K^−1^ mol^−1^) and T is temperature (K). The strong influence of wind speed ($u$) is apparent.

Nevertheless, according to the outcome of the Cosmetics Europe model evaluation [18], Surrey model – CosEU did not perform as well as the CDC and DSkin models when compared with IVPT data, which motivated us to investigate alternative ways of modelling evaporation.

### Evaporation Modelling

This paper presents a new evaporation module that, instead of relying on empirically calculated evaporative mass transfer coefficients, models evaporation as a passive diffusion process driven by the concentration gradient between the air-vehicle interface and the ambient environment. The model simulates the likely evaporation of all ingredients in the vehicle. Evaporation has a dynamic impact on the vehicle. Solvent evaporation leads to an increasing concentration of the active ingredient, which can even precipitate when it exceeds the solubility. On the other hand, if the active ingredient is volatile its evaporation will decrease the concentration in the vehicle. The complex and dynamic changes in the vehicle creates a non-trivial impact on dermal absorption.

The modelling equations are given below. The evaporative flux of an ingredient from the vehicle, ${J}_{evap}$, is given by the Fick’s first law of diffusion in the gas phase above the vehicle:

2 $${J}_{evap}=-{D}_{evap}\times \frac{{M}_{s}-{M}_{a}}{h}$$

where ${M}_{s}$ is the gas-phase molar concentration of the ingredient at the vehicle surface, ${M}_{a}$ is the concentration in the ambient environment beyond the donor chamber of the diffusion cell, and $h$ is the height from the vehicle surface to the top of the wall of the donor chamber (taken as the diffusion cell’s height). We assume that there are no ingredients in the ambient (${M}_{a}=0$), except water for which ${M}_{a}$ can be calculated using the ideal gas law based on humidity. The same ideal gas law can be used to calculate ${M}_{s}$:

3 $${M}_{s}=\frac{P {x}_{l}{ a}_{l}}{R T}$$

where $P$ is the vapour pressure of the pure ingredient, ${x}_{l}$ is its mole fraction in the liquid vehicle and ${a}_{l}$ is the activity coefficient to account for non-ideal liquids (all vehicles assumed to be ideal liquids, (${a}_{l}=1$) in the case study reported in this paper); $T$ is the temperature and $R$ the gas constant.

The diffusivity of a molecule in the air, ${D}_{evap}$, can be obtained from many physical chemistry manuals or calculated through theoretical equations. Two examples are Maxwell–Stefan and Chapman-Enskog [30], which require complex interaction parameters. The Stokes–Einstein equation describes diffusion through a fluid and uses accessible physiochemical parameters to calculate the diffusion coefficient. We compared the results of the Stokes–Einstein equation with both Chapman-Enskog results and experimental values taken from [30], with little variation seen between the three methods for the chemicals tested in this paper. The results reported in this paper are based on D_evap_ calculated using the Stoke-Einstein equation below:

4 $${D}_{evap}=\frac{{K}_{b}\times T}{6 \pi \eta r}$$

where ${K}_{b}$ is the Boltzmann constant, $\eta$ is the dynamic viscosity of air, and $r$ is the radius of the molecule.

### Integration of Evaporation with PBPK Model

To model the impact of evaporation on dermal absorption, the evaporation model has been integrated into the PBPK model developed at Surrey University [18–25]. The integration of evaporation with PBPK modelling considered both solvent and solute volatiles. Volatile evaporation will change the volume of the vehicle ($V$) and this is modelled through the following mass balance:

5 $$\frac{dV}{dt}=-\sum \frac{{J}_{evap} A}{ \rho }$$

where the summation is with respect to all volatile ingredients in the vehicle and $\rho$ is the density of the ingredient; $A$ is the application area. Here again, we assume that the vehicle can be approximated well as an ideal solution to calculate its volume change. In addition, the volume change due to skin permeation is considered negligible.

Evaporation of a volatile will lead to the dynamic change of its mass balance in the vehicle. The overall mass balance of a volatile in the vehicle due to both evaporation and skin penetration is thus given by

6 $$\frac{d(VC)}{dt}=-{J}_{evap}A -{J}_{skin}A$$

Noting

7 $$\frac{d\left(VC\right)}{dt}=V\frac{dC}{dt}+C\frac{dV}{dt}$$

Equation (6) can be rewritten as follows:

8 $$V\frac{dC}{dt}=-{J}_{evap}A+C\sum \frac{{J}_{evap} A}{ \rho }-{J}_{skin}A$$

where ${J}_{skin}$ is the skin permeation flux computed using our existing PBPK model.

This integrated evaporation PBPK model has been implemented in Python 3.8.8, by first including the new evaporative flux calculations in the vehicle compartment of our previous PBPK model, and then coupling it with the rest of the PBPK model as given in the above equations. Open-source code is available on Github: https://github.com/bendeacon/Evaporation. The integrated model is referred to as “PBPK-E” in this paper. It is worth noting that the parameters used in this study were calculated as detailed above without fitting to the experimental data; our model was used in a fully predictive method.

### Model Validation and Application

The Cosmetics Europe ADME (Absorption, Distribution, Metabolism, Excretion) Task Force conducted several projects to measure relevant parameters and help predict the penetration, distribution, and bioavailability of topically applied cosmetic ingredients. Of particular interest to our research is Hewitt *et al.,* 2020 [26] who published IVPT finite dose penetration data for 56 cosmetically relevant chemicals, of which 23 are volatiles. Attempts have been made previously (Gregoire *et al*., 2021) to model the dataset using six in-silico models, including the Surrey model – CosEU*.* Three of these models have simulated evaporation. As discussed above, the models were compared with published experimental data [18] with varying agreements.

Here the newly developed PBPK-E model has been applied to remodel the datasets, focusing on volatile chemicals. To determine volatility, we used the mass balances of permeants reported by Hewitt *et al.*, 2020 [26]. A mass balance, which is the percentage recovery of the test chemical, below 90% was considered indicative of evaporation occurring in the experiment, resulting in 28 chemicals. Grègoire *et al.,* 2019 [18] have reported the vapour pressure from two source*s,* EPA dashboard and the ChemSpider database. However, when checking the vapour pressure against literature values five chemicals had reported vapour pressures significantly deviating from predictions using TEST and OPERA 2.6, and hence were classified as outliers due to the uncertainty, these predictions may have been updated on the EPA dashboard since Grégoire’s publication. The remaining 23 chemicals are listed in Table I, with further details such as experimental mass balance reported in the supplementary information.

**Table I:** The 23 Volatile Permeants Considered in this Study and the Relevant Physiochemical Parameters Have Been Included. A – Taken from the EPA Dashboard (https://comptox.epa.gov/dashboard), B – taken from Grègoire *et al*., 2019, [18]. Several Chemicals have been Applied in Ethanol or Tested in Both Ethanol and PBS, where this is the Case the Vehicle has been Indicated in Brackets after the Chemical, PBS is the Standard Solvent Used

	CAS number	MW ^A^	Log K_ow_^A^	Vapour Pressure (Pa) ^B^
4-Tolunitrile	104–85-8	117.15	1.58	41.72
Acetophenone	98–86-2	120.15	0.20	52.90
Aminophenol	95–55-6	109.13	0.62	0.01
Benylidene Acetone	122–57-6	146.19	2.07	1.65
Benzophenone (Ethanol)	119–61-9	182.22	3.18	0.26
Benzophenone (PBS)	119–61-9	182.22	3.18	0.26
Cinnamaldehyde	14,371–10-9	132.16	1.90	5.12
Diethylmaleate	141–05-9	172.18	0.82	14.00
Dimethyl fumarate	624–49-7	144.13	1.74	40.00
Dimethyl phthalate	131–11-3	194.19	1.58	0.41
Ethylhexyl Acrylate	103–11-7	184.28	4.20	23.70
Eugenol	97–53-0	164.20	2.27	3.02
Geraniol (Ethanol)	106–24-1	154.25	3.56	4.00
Geraniol (PBS)	106–24-1	154.25	3.56	4.00
Isoeugenol	97–54-1	164.20	3.04	1.60
Methyl Methane sulfonate	66–27-3	110.13	0.74	55.20
Methylisothiazolinone	2682–20-4	115.15	-0.10	4.13
Naphthalene	91–20-3	128.17	3.30	11.30
Nitrobenzene	98–95-3	123.11	1.85	32.70
Propylparaben (Ethanol)	94–13-3	180.20	3.04	0.24
Tetramethyl thiuram disulfide	137–26-8	240.42	1.73	0.0023
Thioglycolic Acid	68–11-1	92.11	0.09	11.60
Vanillin	121–33-5	152.15	1.19	0.02

The PBPK-E model was applied, with a simulation time of 24 h, to predict against the cutaneous distribution and receptor fluid (RF) kinetics data reported by the Cosmetics Europe task force [26]. The diffusion, partition and vapour pressure parameters used in this study were calculated from theoretical and/or QSPR equations as detailed above without fitting to the experimental data. Furthermore, a comparison to the three dermal models with evaporation from Grègoire *et al.,* 2021 [18] was performed, to illustrate the improvement of prediction of the PBPK-E model in terms of the coefficient of determination (R^2^) of the predicted result vs. experimental results for each skin compartment. The three models (CDC, DSkin and Surrey model – CosEU*)* taken from Grègoire *et al.,* 2021 [18] include evaporation, modelled using wind speed as the main factor affecting evaporation shown in Eq. (1). Simulations using DSkin and *Surrey model –* *CosEU* were performed by the developers of the respective model and the CDC simulations were run by modelling experts. For further details on the simulations the reader is referred to the supplementary information provided by Grègoire *et al.,* 2021 [18].

## Results

**Fig. 1:**
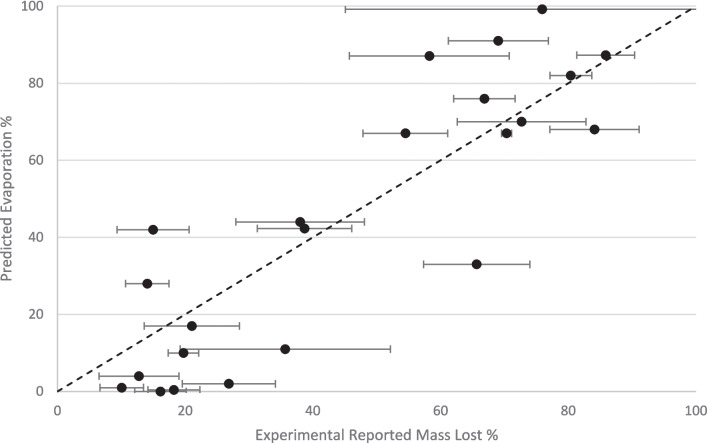
Comparison of PBPK-E model predictive evaporation with experimental mass loss and error bars reported in [26], where the experimental values, from Hewitt *et al*., 2020 [26], are reported as the mean of three experiments ± standard deviation. R^2^ = 0.76 (n = 23). The dashed line indicates perfect agreement.

The PBPK-E model has been applied to simulate the Cosmetics Europe IVPT study [26]. First, the model’s prediction of the 23 volatiles has been compared with the mass imbalance of the reported data as shown in Fig. 1. The predicted evaporation results agreed well with the reported mass loss (R^2^ = 0.76, n = 23).

Table II shows the predicted distribution of the permeants in each compartment compared with the Cosmetics Europe data: with and without evaporation.

**Table II:** Comparison of Model Prediction with Experimental Data, in Terms of Percentage Accumulated of Permeants in each Compartment at the end of 24 h of Exposure. Where the PBPK Model is the Existing Dermal Model without Evaporation [18–25]. The PBPK-E Model is the Dermal Model with an Evaporation Module

Compartment	R^2^ PBPK model [23]	R^2^ PBPK-E model
Atmosphere (mass loss)	-	0.76
Vehicle	0.19	0.55
Stratum Corneum (SC)	0.15	0.11
Viable Epidermis	0.01	0.00
Dermis	0.00	0.01
Receptor Fluid (RF)	0.19	0.68
Dermal Delivery*	0.15	0.60

* ‘Dermal delivery’ refers to the combination of the viable epidermis, dermis, and RF

**Fig. 2:**
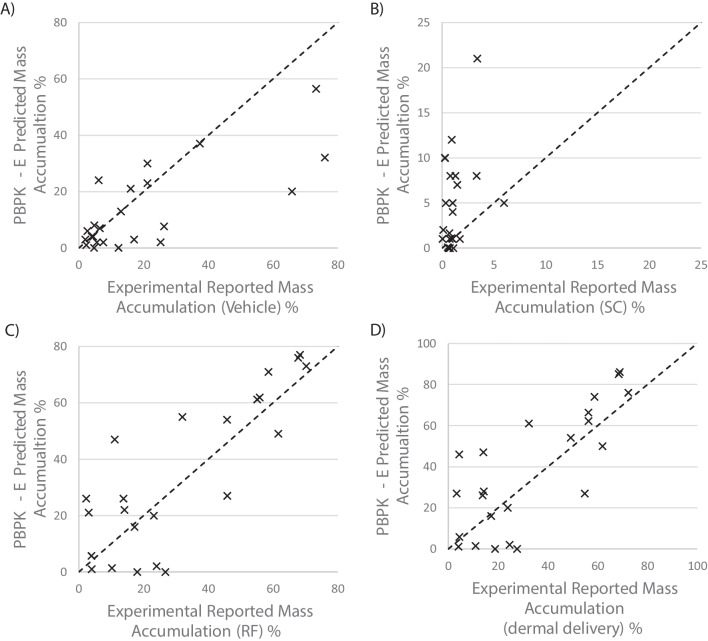
Hewitt data vs. PBPK-E model deposition in four compartments. **A** Comparison in the vehicle (R^2^ = 0.55). **B** Comparison in the SC (R^2^ = 0.11). **C** Comparison in the RF (R^2^ = 0.66). **D** Comparison with the dermal delivery (R^2^ = 0.60). The dashed line indicates perfect agreement.

The corresponding scatter plots to Table II are given in Fig. 2. Figure 2 shows the agreement between the PBPK-E model predictions and the published IVPT data from Hewitt *et al.*, 2020 [26] in each compartment. This shows the variance of agreement in each compartment with vehicle, RF and dermal delivery being the strongest, the SC shows an ok agreement between the PBPK-E model and experiment. The viable epidermis and dermis have been excluded from the Figure due to poor correlation between prediction and experimental data.

**Fig. 3:**
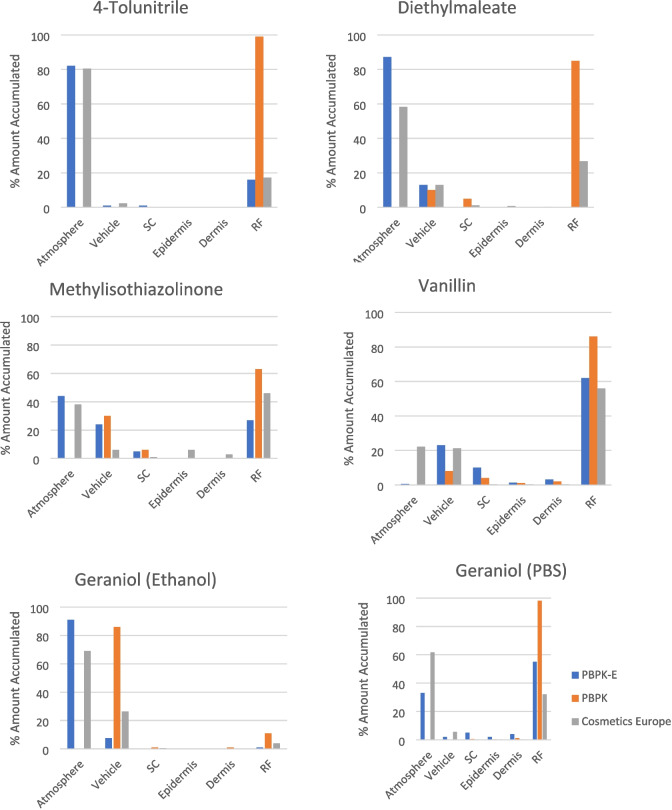
Distribution of 4-Tolunitrile, Diethylmaleate, Methylisothiazolinone, Vanillin, Geraniol (Ethanol), and Geraniol (PBS) after 24 h, as predicted by the two models against experimental data. The Cosmetics Europe data is experimental *in vitro* data reported by Hewitt *et al*., 2020 [26], measured over 24 h in the receptor fluid. Where the PBPK model is the existing dermal model without evaporation [18–25]. The PBPK-E model is the dermal model with an evaporation module.

**Fig. 4:**
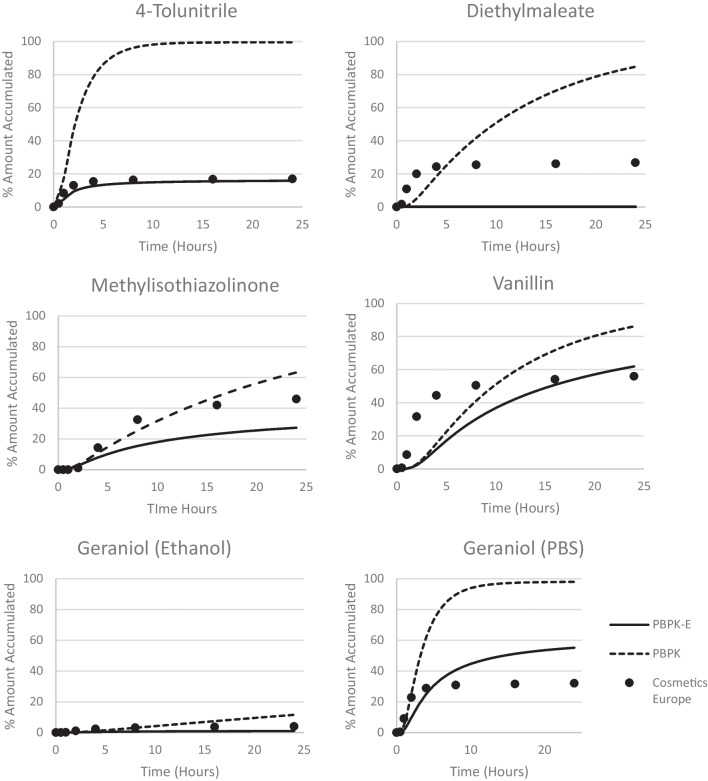
RF kinetics for the six chosen chemicals: 4-Tolunitrile, Diethylmaleate, Methylisothiazolinone, Vanillin, Geraniol (Ethanol), and Geraniol (PBS). The Cosmetics Europe data is experimental *in vitro* data reported by Hewitt *et al*., 2020 [26], measured over 24 h in the receptor fluid. Where the PBPK model is the existing dermal model without evaporation [18–25]. The PBPK-E model is the dermal model with an evaporation module.

**Fig. 5:**
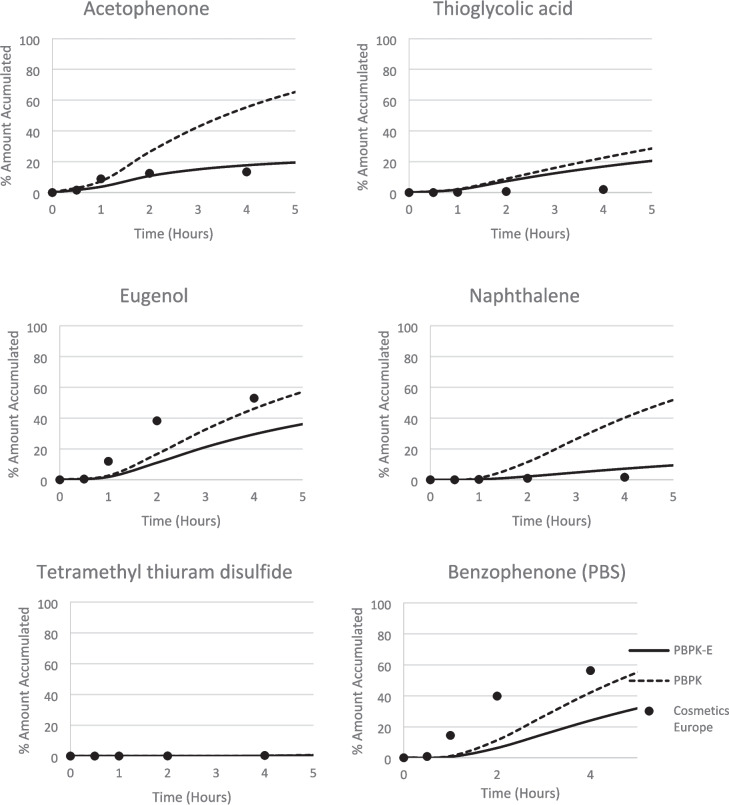
RF kinetics for six chemicals: Acetophenone, Thioglycolic acid, Eugenol, Naphthalene, Tetramethyl thiuram disulfide, and Benzophenone (PBS). The Cosmetics Europe data is experimental *in vitro* data reported by Hewitt *et al*., 2020 [26], measured over 5 h in the receptor fluid to show the initial kinetic phase. Where the PBPK model is the existing dermal model without evaporation [18–25]. The PBPK-E model is the dermal model with an evaporation module.

Further details of the predicted kinetic change of cutaneous distribution and receptor fluid (RF) in comparison with reported experimental data are plotted in Fig. 3 and Fig. 4. 4-Tolunitrie, methylisothiazolinone, geraniol (applied in both ethanol and PBS solvent), diethylmaleate and vanillin were chosen to represent a range of physiochemical properties and model-data agreement. Furthermore, the initial kinetics are shown, Fig. 5 for a further six chemicals to explore the behaviour of the two models at early time steps.

The RF kinetics, predicted by models and compared with experimental data, for the remaining 17 chemicals are given in the supplementary information. The input data required to recreate the simulations is included in the supplementary information.

Grègoire *et al*., 2021 [18] has reported six dermal models, of which three include evaporation as a function of wind speed: CDC, DSkin, and Surrey model – CosEU. A comparison between the three models with evaporation from Grègoire *et al.,* 2021 and the PBPK and PBPK-E models has been performed in Table III. The PBPK-E model exhibits a high R^2^ value in the atmosphere and outperforms the three models presented in Grègoire *et al*., 2021 for both atmospheric and RF compartments.

**Table III:** Comparison of R^2^ Values for Different Dermal Models. The R^2^ is Between the model and the Hewitt Experimental for the Atmosphere Compartment and the RF at the end of 24 h of Exposure. A—R^2^ Published in Grègoire *et al*., 2021 [18], B—R^2^ Values Calculated in this Paper. Where the PBPK Model the Existing Dermal Model without Evaporation [18–25]. The PBPK-E model is the Dermal Model with an Evaporation Module

Model	Atmosphere R^2^	Dermal Delivery R^2^
CDC ^A^	0.59	0.58
DSkin ^A^	0.69	0.60
Surrey model – CosEU ^A^	0.61	0.23/0.29*
PBPK-E model ^B^	0.76	0.60
PBPK model ^B^	-	0.15

* Grègoire *et al*., 2021 [18] has published two values, using the Surrey model – CosEU with a nominal dose produces an R^2^ = 0.23, adjusting the dose to account for evaporation produces an R^2^ = 0.29

## Discussion

The PBPK-E model’s predictions of evaporation of the permeants align well with atmospheric data, producing a high R^2^ value (R^2^ = 0.76, n = 23). This indicates that the approach to model evaporation through the use of vapour pressure as the main factor is a valid method. This paper has only considered volatile chemicals hence the model may not apply to non-volatile chemicals or may decrease the accuracy of the results when applied to non-volatile chemicals. Where in this study to determine volatility, we used the mass balances of permeants reported by Hewitt *et al.*, 2020 [26]. A permeant with a mass balance below 90% was considered volatile, a mass balance above 90% the permeant was assumed to not be volatile.

The cumulative amounts in the vehicle and the RF are predicted satisfactorily, with an R^2^ of 0.55 and 0.68, respectively. However, the amount remaining in the SC is generally over-predicted, and those in the viable epidermis and dermis compartments were poorly predicted. It was well recognised that quantifying the amount of permeant in individual skin layers is challenging and the existing methods (e.g., tape stripping) are subject to significant variabilities. Furthermore, the models reported in this work assumed that permeants have the same properties (diffusion and partition) in the viable epidermis as in the dermis, due to the lack of experimental information to differentiate them [31].This assumption is justifiable for overall dermal delivery as the stratum corneum is the primary barrier; however, it is not accurate if the intention is to predict the amount remaining in these skin layers.

Among the 23 chemicals examined, varying levels of agreement between the model and experiment were observed. From the three chemicals shown, vanillin and 4-tolunitrile demonstrated good endpoint agreement (the distribution of the chemical at 24 h post exposure) across all compartments; diethylmaleate's had a large overestimation in the atmosphere compartment, which may stem from uncertainties in the source of vapour pressure data. These chemicals are representative of the larger dataset, and the results are included in the supplementary information. The best predicted chemicals (4-tolunitrile, methyl methane sulfonate, nitrobenzene and naphthalene (applied in ethanol) in both the atmosphere and the receptor fluid, tend to be very volatile with a VP > 20 and applied in PBS. Chemicals that are not in agreement with experiment appear to be less volatile with a VP between 0 and 10. Chemicals applied in ethanol have a strong agreement with atmosphere predictions but appear to have a weaker agreement in the RF. MW and log K_ow_ do not appear to influence the agreement as strongly as VP, with the PBPK-E model strongly predicting chemicals in the MW range 90 to 200 Da and log K_ow_ between 0 to 4.

For the RF kinetics, visual comparisons are performed of the results between the two models (PBPK and PBPK-E) and the experimental data. A range of agreement over the course of 24 h have been shown. Vanillin’s RF kinetics from the PBPK-E model has an agreement with kinetics over 24 h to the experimental data but demonstrates a high endpoint agreement; both kinetics and endpoint agreement are improved with the addition of evaporation. 4-Tolunitrile demonstrated excellent RF kinetics agreement across the 24 h, with significant improvement from the PBPK to the PBPK-E model. Diethylmaleate has a poor agreement for both kinetics and endpoint agreement in both the PBPK and PBPK-E model. This may be a result of uncertainty in predicted parameters, or uncertainty in the experimental measurement. From the RF kinetics and cutaneous distribution graphs shown in the supplementary information it is clear that the PBPK-E model produces strong agreement for the RF kinetics and the cutaneous distribution at 24 h. During the initial kinetics phase, the two models are highly variable in their ability to recreate the experimental kinetics. The agreement for initial kinetics for the first 5 h is slightly weaker for the PBPK-E model where the presence of evaporation increases the lag time and lowers the amount permeated into the RF during this initial stage. To note is that the experimental cutaneous distribution is only reported at 24 h and the models cannot be compared to experimental distribution data after 5 h, the RF kinetics are reported for the full-time frame allowing for the comparison of early phase kinetics.

There is a significant improvement of the PBPK-E model over the PBPK model without evaporation. This highlights evaporation is needed to obtain accurate predictions of IVPT dermal absorption data under unoccluded, finite dose conditions.

The comparison performed between the PBPK model, PBPK-E model and the models presented in Grègoire *et al*., 2021 [18] evaluates vapour pressure and wind speed as the main factors impacting evaporation. All four of the models that consider evaporation perform significantly better than the PBPK model without evaporation. The PBPK-E model presented uses the vapour pressure assumption, and has been shown to have a better agreement with the experimental data than the models presented in Grègoire *et al*., 2021 [18]. This may be a result of the apparatus used in the IVPT experiments, where a donor chamber that is tall relative to its cross-sectional area will reduce the impact wind speed will have on the formulation [17]. Consideration of wind speed may become necessary for outdoor conditions and *in-vivo* experiments.

For the applicability domain of the PBPK-E model, MW from 90 to 240 Da, log K_ow_ from – 0.1 to + 4.2 and a vapour pressure between 0.001 Pa and 56 Pa. The PBPK model has an applicability domain of MW from 100 to 500 Da, log K_ow_ from – 1 to + 8. The PBPK-E domain is likely larger, due to the applicability domain of the PBPK model; however, the PBPK-E model has been applied to the Hewitt dataset which has a small chemical range. Furthermore, both the PBPK and PBPK-E model have simulated leave-on scenarios where the vehicle is applied to the skin for the duration of the experiment, which is representative of the cosmetic chemical application in Hewitt *et al.,* 2020 [26].

The PBPK-E model has been applied to single solvent systems namely PBS, ethanol and acetone. The PBPK model has tested a larger range of tested solvents including olive oil, acetone, ethanol, methanol, PBS, and dichloromethane. Both models simulate both aqueous and non-aqueous solvents given the permeant-vehicle solubility. As shown in the methods, all vehicles are assumed to be ideal in their behaviour, non-ideal behaviour will reduce the amount evaporated and delivered into the skin due to interactions in the vehicle.

## Conclusion

This paper presented a mechanistic model for simulating the impact of evaporation of volatile permeants on their dermal absorption. Using vapour pressure as a reliable indicator of chemical volatility under laboratory evaporation scenarios, the rate of evaporative loss has been predicted without empirical parameter fitting. However, parameter calibration could improve the accuracy of the RF delivery. The proposed PBPK-E model demonstrates strong agreement with the experimental data. Moreover, our model demonstrates comparability with the models presented in Grègoire *et al*., 2021, which underscores the PBPK-E models’ potential use in pharmaceutical and toxicological research. The predictive nature of the model benefits novel and hazardous chemicals, which can be simulated without the need for experimental data. The study emphasises the critical role of evaporation in dermal absorption modelling, particularly recreating finite dose, unoccluded conditions. The in-silico model presented in this work is being considered for inclusion into US EPA’s httk model [32] for assessment of dermal exposure.

## Supplementary Information

Below is the link to the electronic supplementary material.

Supplementary file1 (PDF 88 KB)

Supplementary file2 (PDF 282 KB)

Supplementary file3 (XLSX 53 KB)

## Data Availability

Data generated in this study is available with the evaporation model in the GitHub repository: https://github.com/bendeacon/Evaporation. The full results and input values are included in the supplementary information.

## Abbreviations

ADME: Absorption, distribution, metabolism, excretion
$${a}_{l}$$: Activity coefficient
$A$: Area of application (m^2^)
K_b_: Boltzmann constant (J K^−^^1^)
${M}_{a}$: Concentration in the ambient environment
C: Concentration of the active ingredient (mol m^−^^3^)
$$\rho$$: Density (mol m^−^^3^)
D_evap_: Diffusion coefficient (m^2^ s^−^^1^)
η: Dynamic viscosity of air (Pa s)
PBPK-E: Physiologically based pharmacokinetic model with Evaporation
J_evap_: Evaporation flux (mol m^−^^2^ s^−^^1^)
K_evap_: Evaporation mass transfer coefficient (m s^−^^1^)
J_skin_: Flux into the skin (mol m^−^^2^ s^−^^1^)
R: Gas constant (J K^−^^1^ mol^−^^1^)
M_s_: Gas-phase molar concentration
h: Height from the liquid surface to the top of the wall of the donor chamber (m)
IVPT: *In vitro* permeation test
${x}_{l}$: Mole fraction in the liquid vehicle
MW: Molecular weight (Da)
PBPK: Physiologically based pharmacokinetic model
PBS: Phosphate buffered solution
QSPR: Quantitative structure property relationship
r: Radius of the molecule (m)
RF: Receptor fluid
SC: Stratum corneum
T: Temperature (°K)
V: Volume (m^3^)
P: Vapour pressure (Pa)
u: Wind speed (m s^−^^1^)
