# Supplementary material for: Computational Modelling of the Impact of Evaporation on In-Vitro Dermal Absorption
Source: Pharm Res. 2024 Oct 7;41(10):1979–90. doi: 10.1007/s11095-024-03779-y (PMC11530481; doi:10.1007/s11095-024-03779-y)
Supplement: Supplementary file 1 — Supplementary file1 (PDF 88 KB) [file 11095_2024_3779_MOESM1_ESM.pdf]

Cutaneous Distribution for all 23 chemicals.

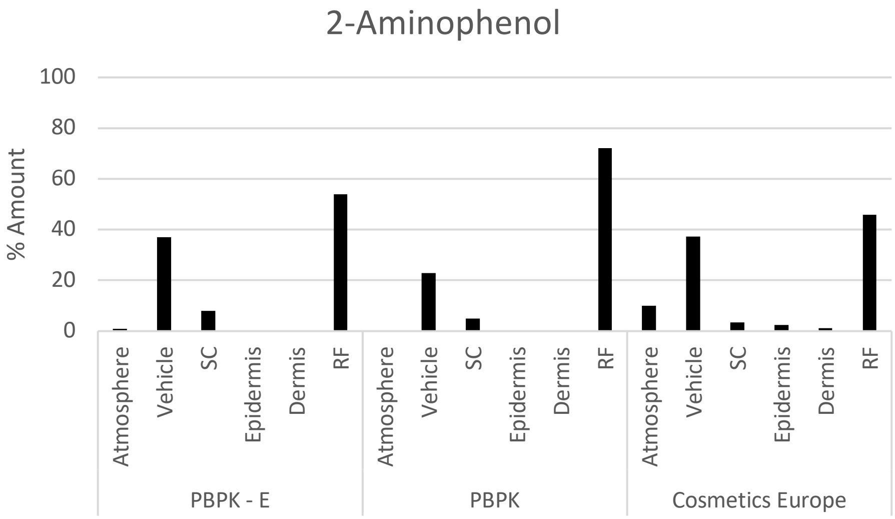

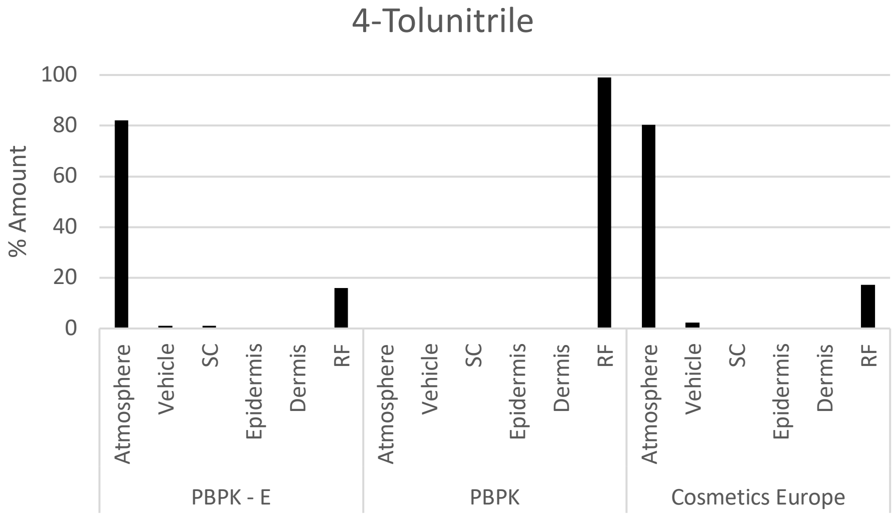

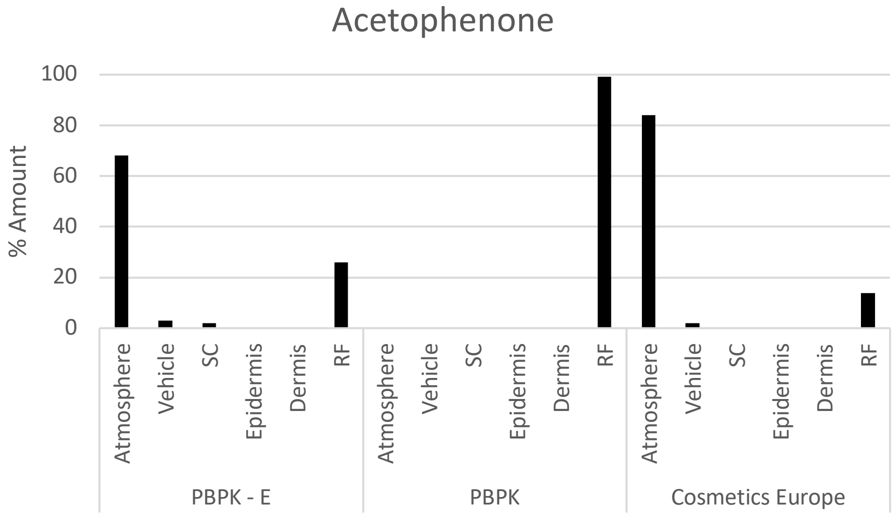

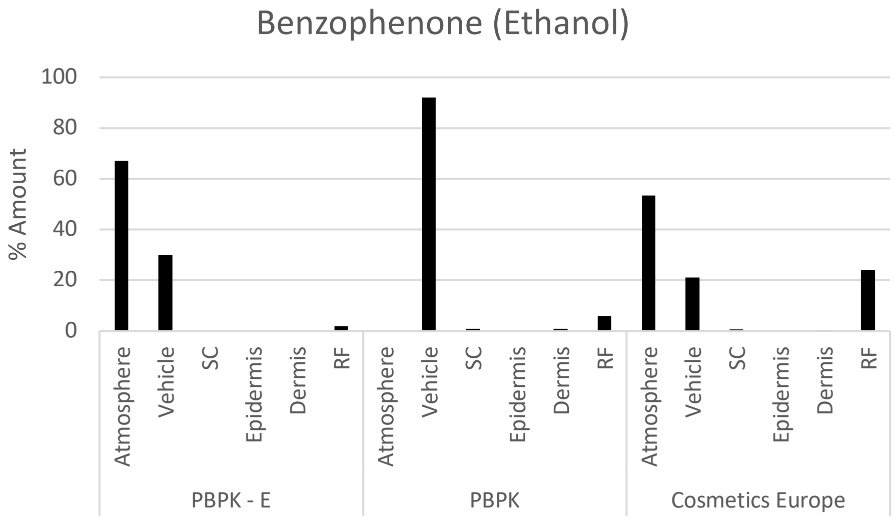
Benzophenone (Ethanol)
% Amount
0
20
40
60
80
100
Atmosphere
Vehicle
SC
Epidermis
Dermis
RF
PBPK - E
PBPK
Cosmetics Europe

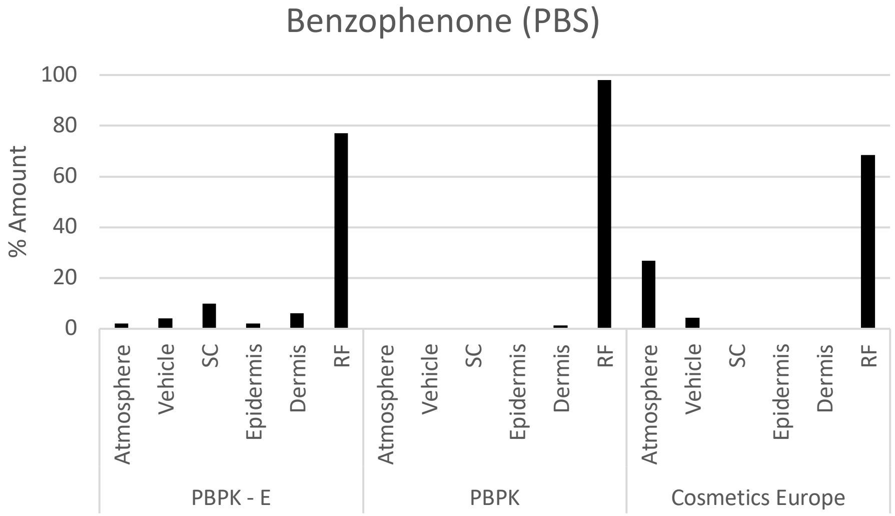
Benzophenone (PBS)
% Amount
0
20
40
60
80
100
Atmosphere
Vehicle
SC
Epidermis
Dermis
RF
PBPK - E
PBPK
Cosmetics Europe

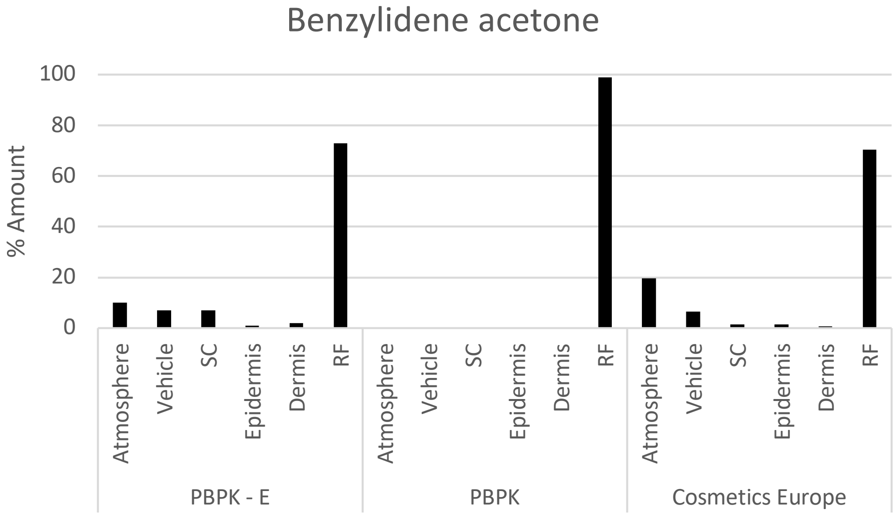
Benzylidene acetone
% Amount
0
20
40
60
80
100
Atmosphere
Vehicle
SC
Epidermis
Dermis
RF
PBPK - E
PBPK
Cosmetics Europe

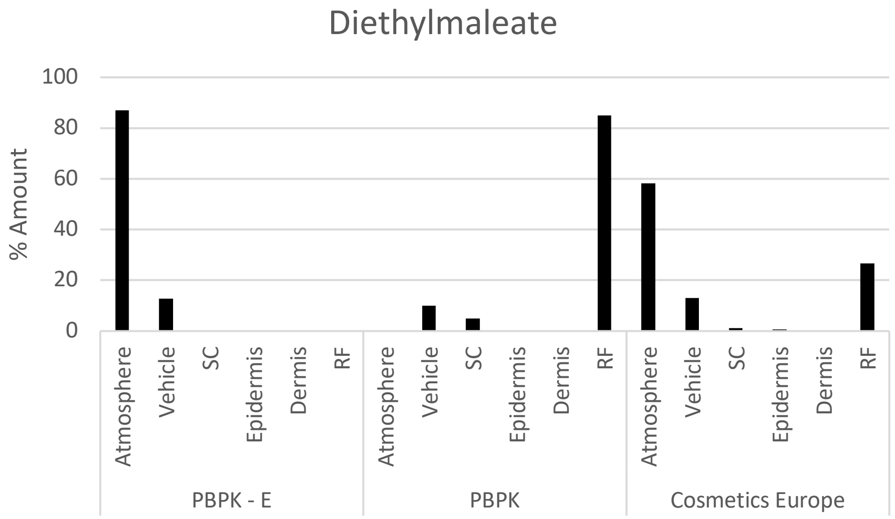
Diethylmaleate
% Amount
100
80
60
40
20
0
Atmosphere
Vehicle
SC
Epidermis
Dermis
RF
PBPK - E
PBPK
Cosmetics Europe

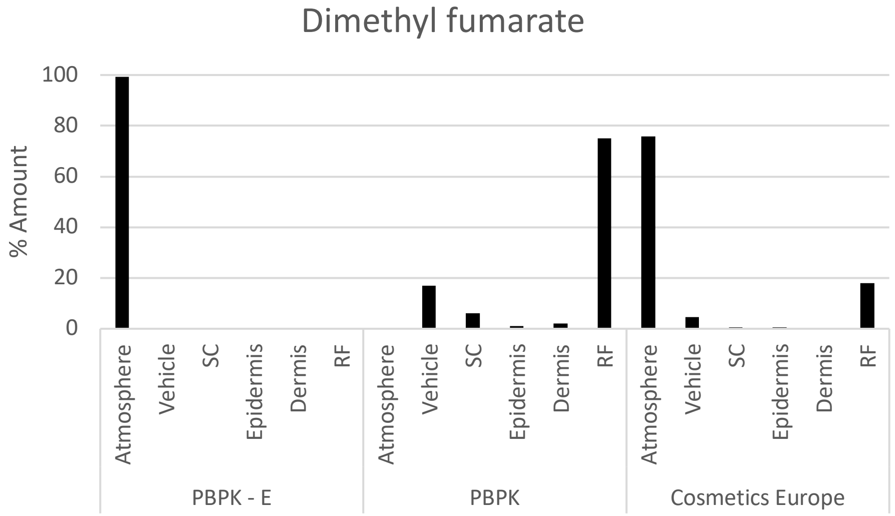
Dimethyl fumarate
% Amount
100
80
60
40
20
0
Atmosphere
Vehicle
SC
Epidermis
Dermis
RF
PBPK - E
PBPK
Cosmetics Europe

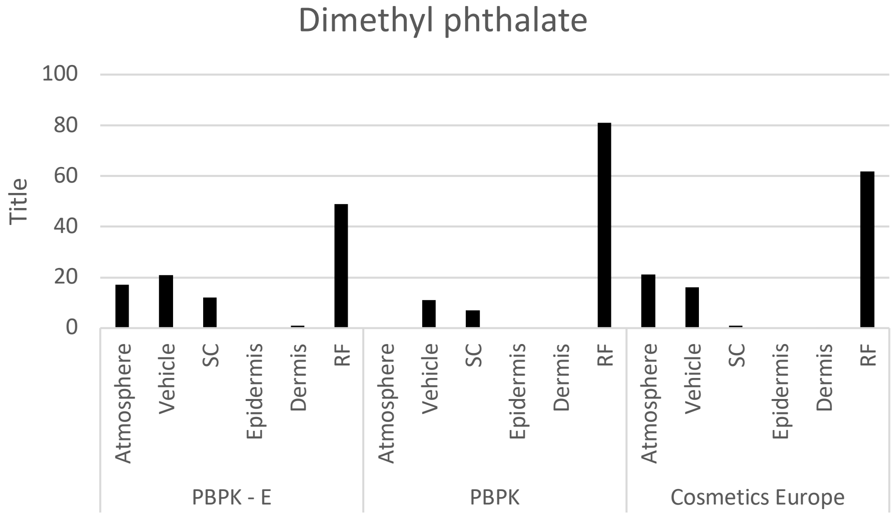
Dimethyl phthalate
Title
100
80
60
40
20
0
Atmosphere
Vehicle
SC
Epidermis
Dermis
RF
PBPK - E
PBPK
Cosmetics Europe

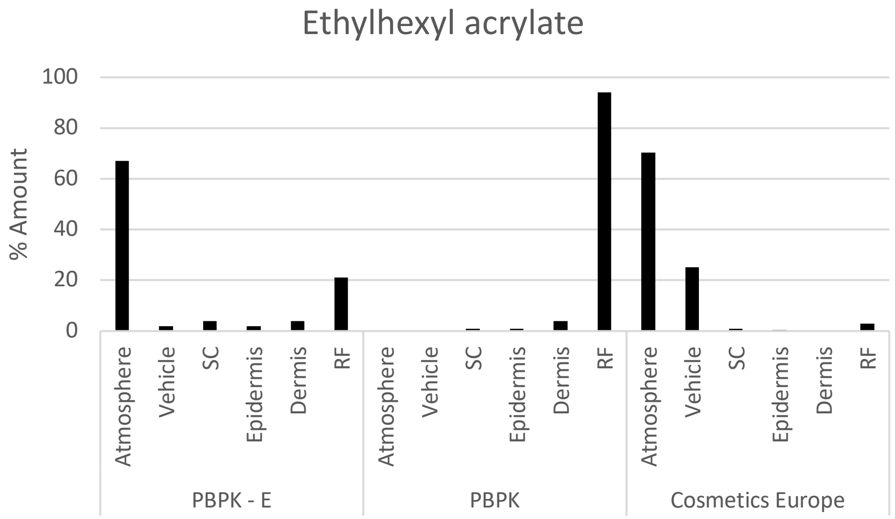
Ethylhexyl acrylate
% Amount
100
80
60
40
20
0
Atmosphere
Vehicle
SC
Epidermis
Dermis
RF
PBPK - E
PBPK
Cosmetics Europe

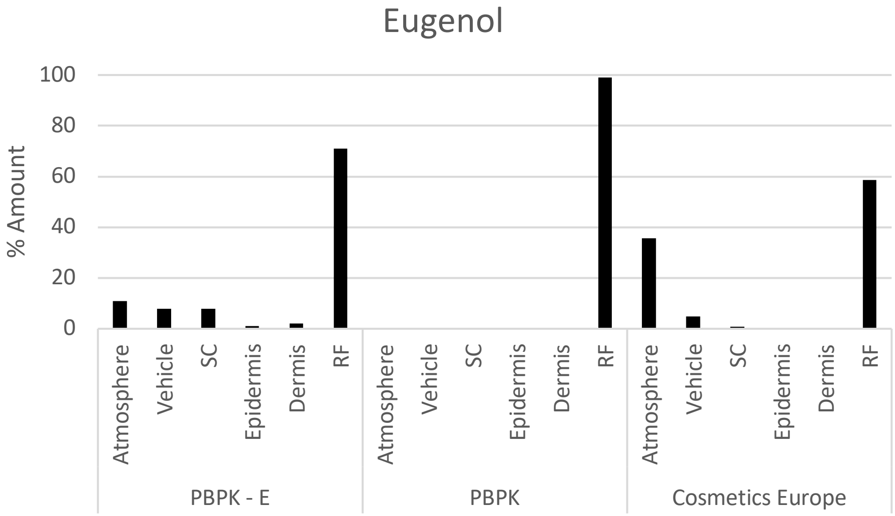
Eugenol
% Amount
100
80
60
40
20
0
Atmosphere
Vehicle
SC
Epidermis
Dermis
RF
PBPK - E
PBPK
Cosmetics Europe

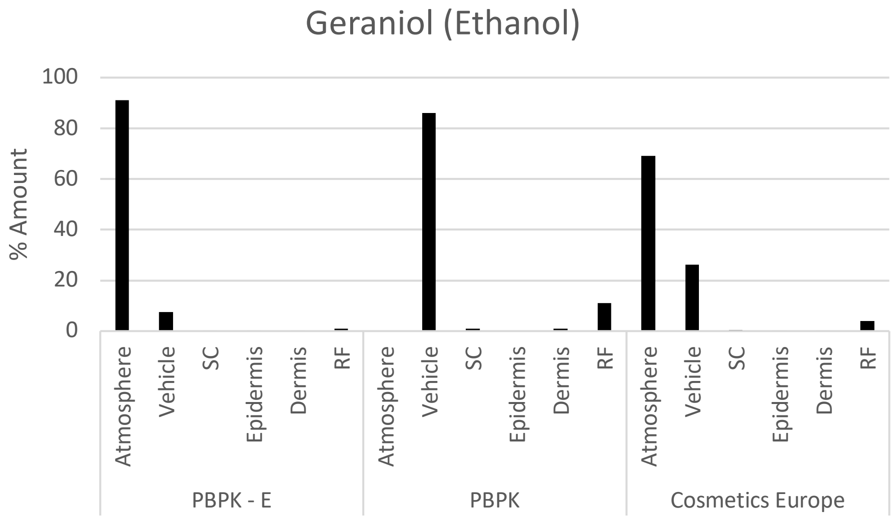
Geraniol (Ethanol)
% Amount
100
80
60
40
20
0
Atmosphere
Vehicle
SC
Epidermis
Dermis
RF
PBPK - E
PBPK
Cosmetics Europe

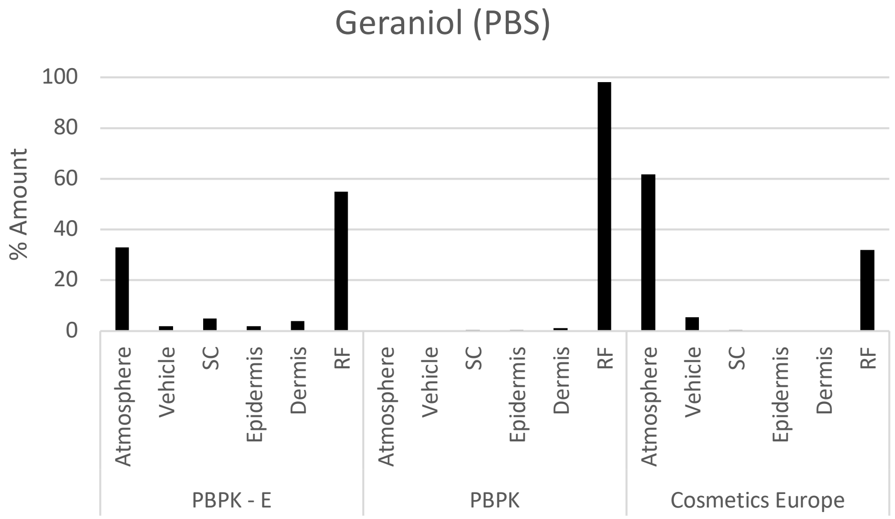
Geraniol (PBS)
% Amount
0
20
40
60
80
100
Atmosphere
Vehicle
SC
Epidermis
Dermis
RF
PBPK - E
PBPK
Cosmetics Europe

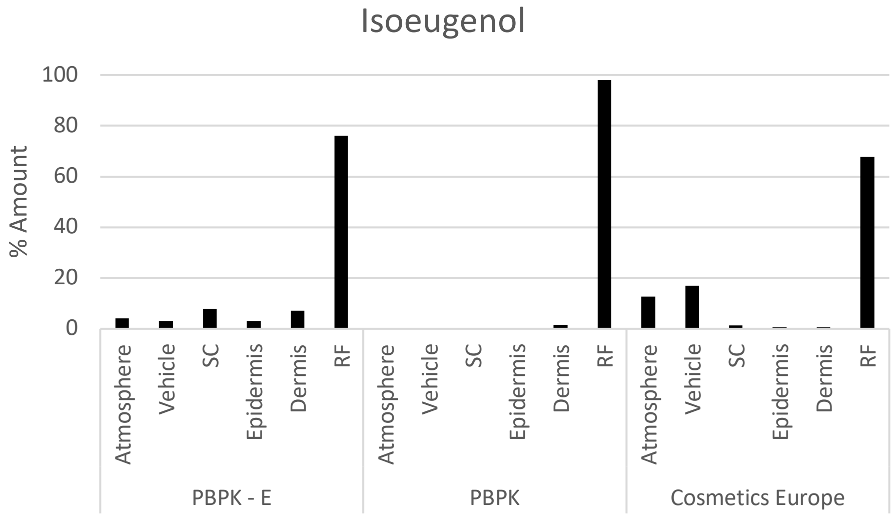
Isoeugenol
% Amount
0
20
40
60
80
100
Atmosphere
Vehicle
SC
Epidermis
Dermis
RF
PBPK - E
PBPK
Cosmetics Europe

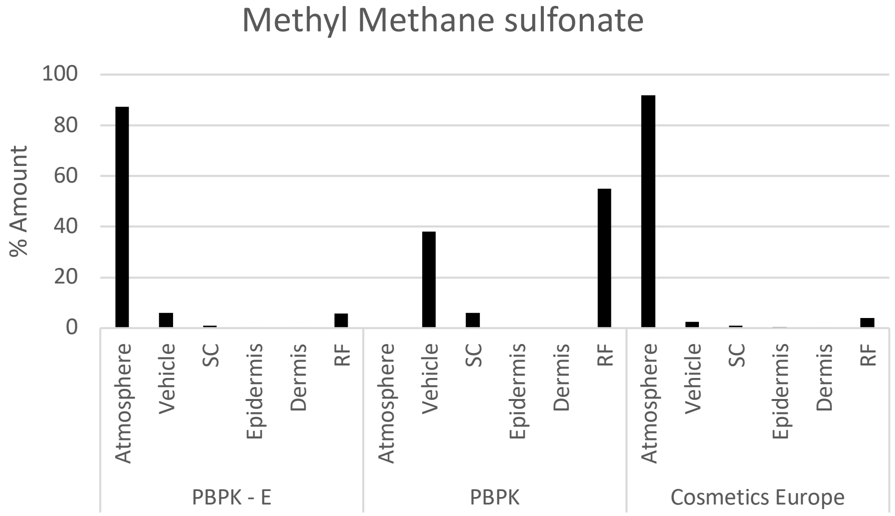
Methyl Methane sulfonate
% Amount
0
20
40
60
80
100
Atmosphere
Vehicle
SC
Epidermis
Dermis
RF
PBPK - E
PBPK
Cosmetics Europe

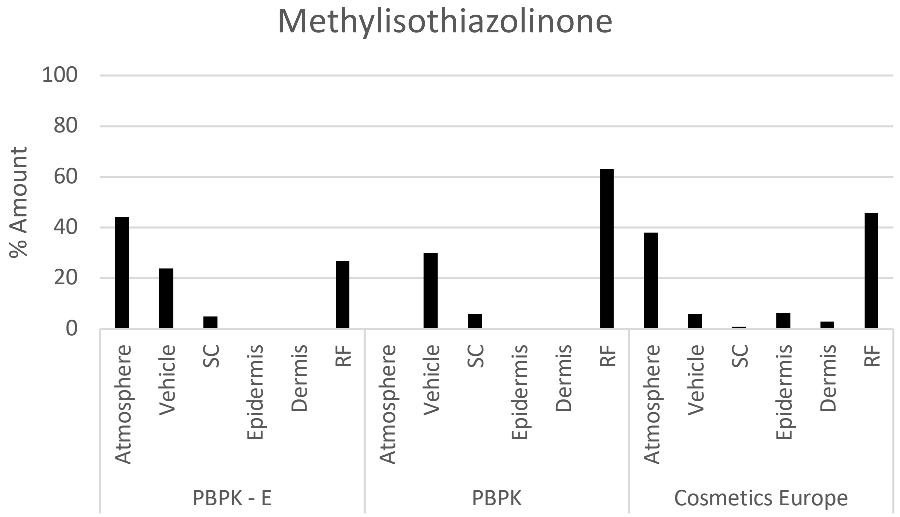
Methylisothiazolinone
% Amount
0
20
40
60
80
100
Atmosphere
Vehicle
SC
Epidermis
Dermis
RF
PBPK - E
PBPK
Cosmetics Europe

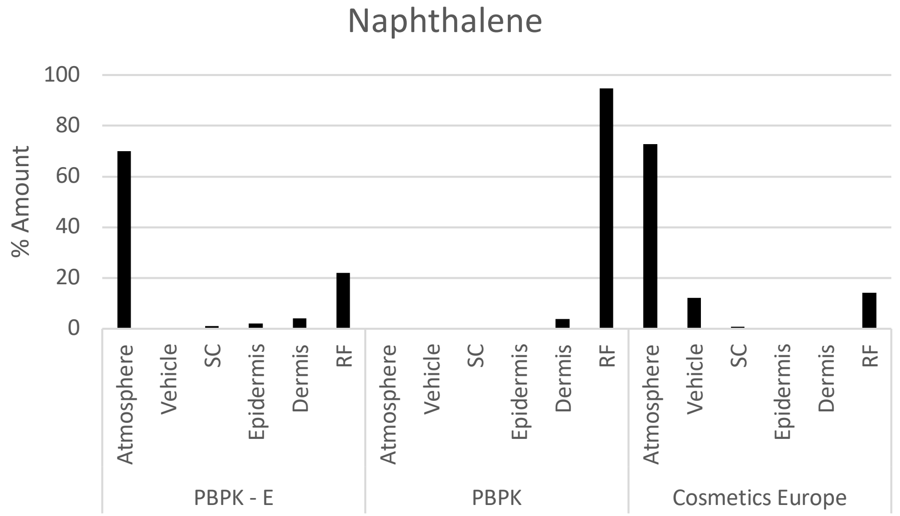
Naphthalene
% Amount
0
20
40
60
80
100
Atmosphere
Vehicle
SC
Epidermis
Dermis
RF
PBPK - E
PBPK
Cosmetics Europe

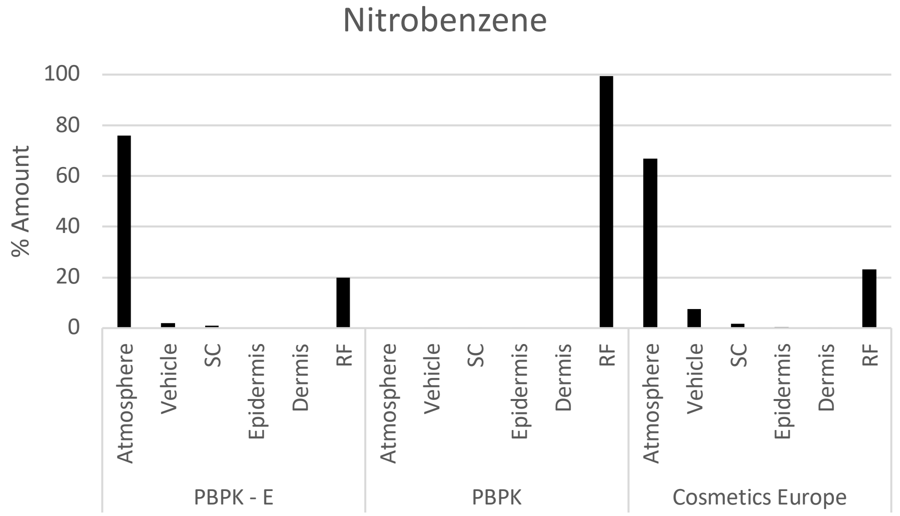
Nitrobenzene
% Amount
0
20
40
60
80
100
Atmosphere
Vehicle
SC
Epidermis
Dermis
RF
PBPK - E
PBPK
Cosmetics Europe

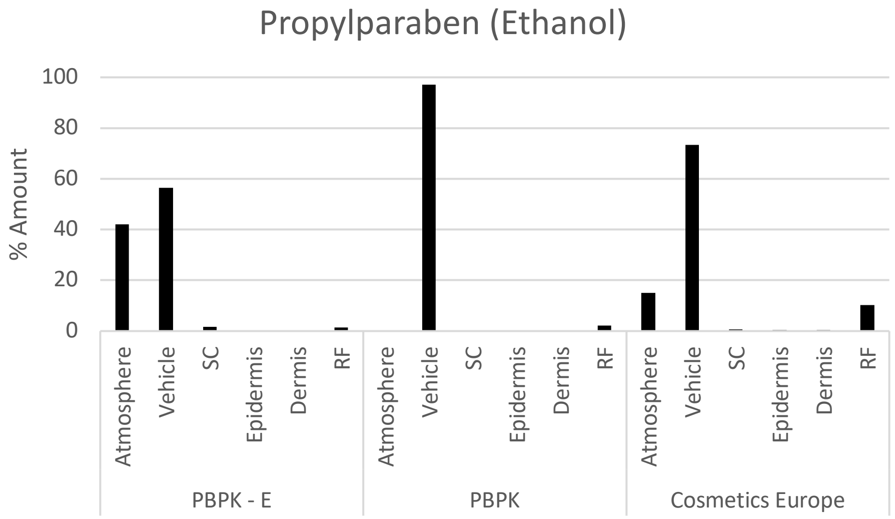
Propylparaben (Ethanol)
% Amount
0
20
40
60
80
100
Atmosphere
Vehicle
SC
Epidermis
Dermis
RF
PBPK - E
PBPK
Cosmetics Europe

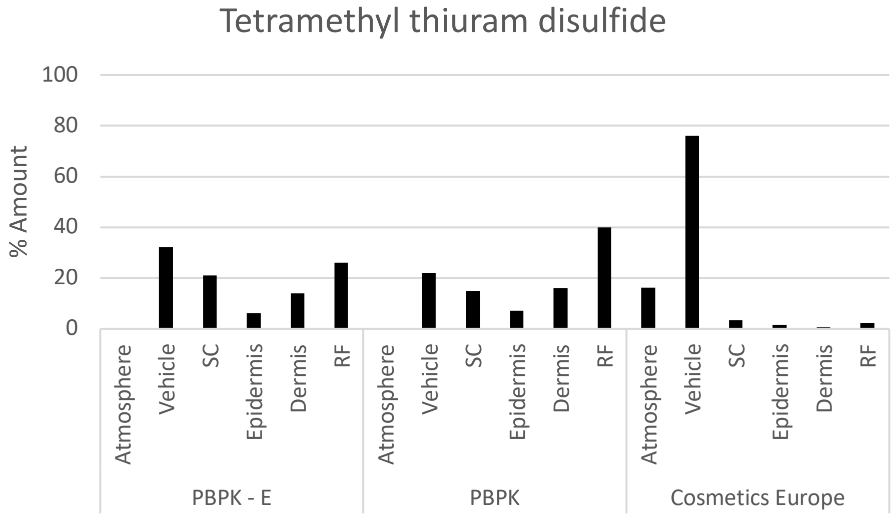
Tetramethyl thiuram disulfide
% Amount
0
20
40
60
80
100
Atmosphere
Vehicle
SC
Epidermis
Dermis
RF
PBPK - E
PBPK
Cosmetics Europe

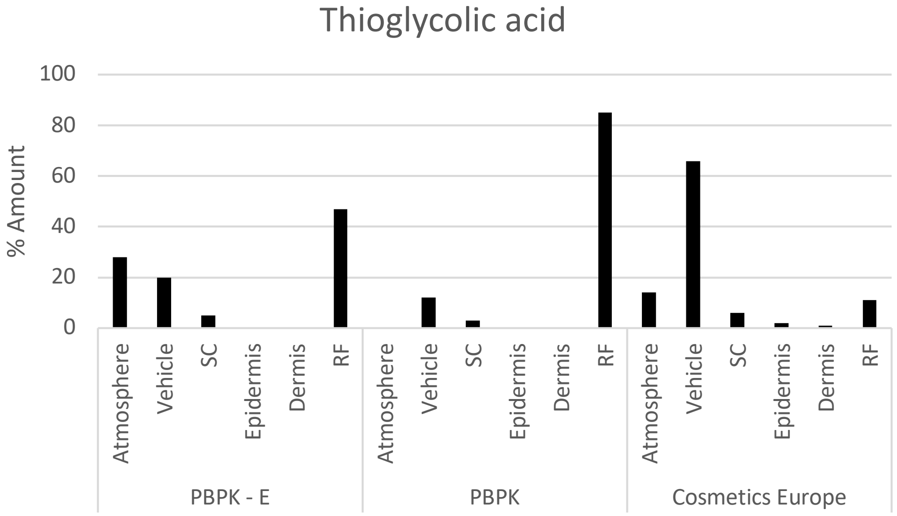
Thioglycolic acid
% Amount
0
20
40
60
80
100
Atmosphere
Vehicle
SC
Epidermis
Dermis
RF
PBPK - E
PBPK
Cosmetics Europe

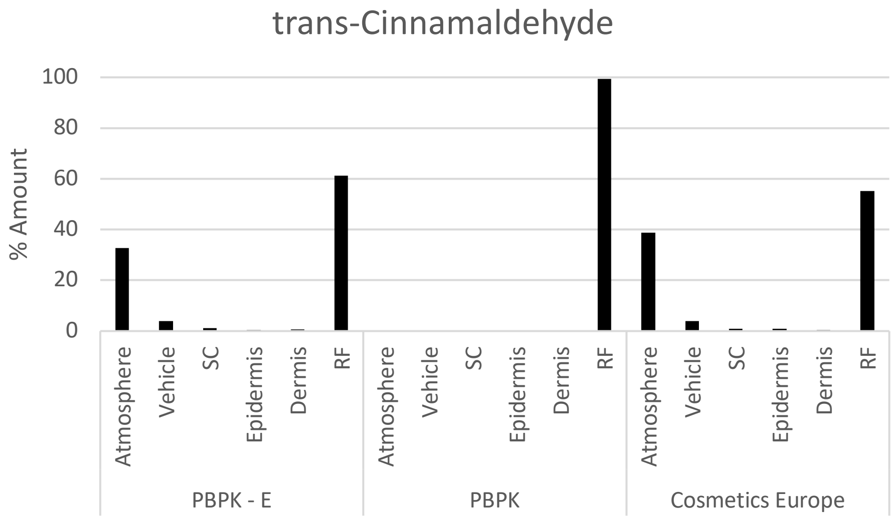
trans-Cinnamaldehyde
% Amount
100
80
60
40
20
0
Atmosphere
Vehicle
SC
Epidermis
Dermis
RF
PBPK - E
PBPK
Cosmetics Europe

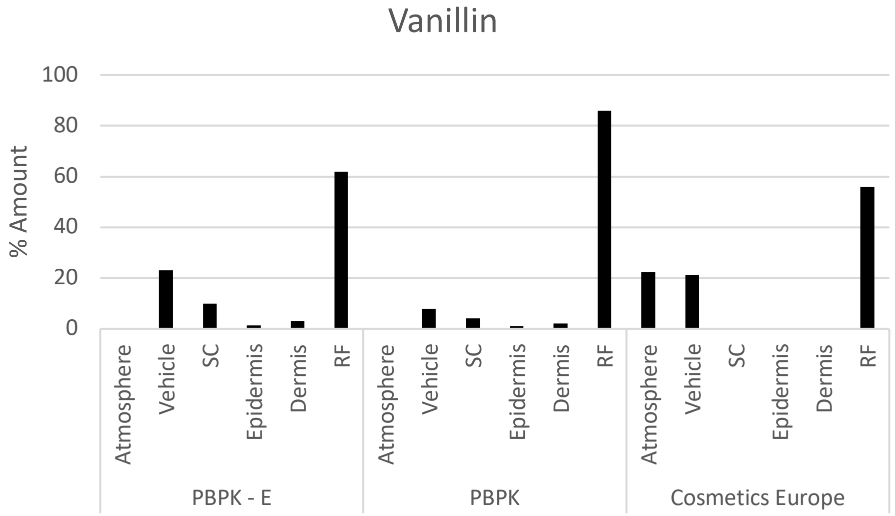
Vanillin
% Amount
100
80
60
40
20
0
Atmosphere
Vehicle
SC
Epidermis
Dermis
RF
PBPK - E
PBPK
Cosmetics Europe
