# Supplementary material for: Computational Modelling of the Impact of Evaporation on In-Vitro Dermal Absorption
Source: Pharm Res. 2024 Oct 7;41(10):1979–90. doi: 10.1007/s11095-024-03779-y (PMC11530481; doi:10.1007/s11095-024-03779-y)
Supplement: Supplementary file 2 — Supplementary file2 (PDF 282 KB) [file 11095_2024_3779_MOESM2_ESM.pdf]

Receptor Fluid Kinetics graphs for all 23 chemicals.

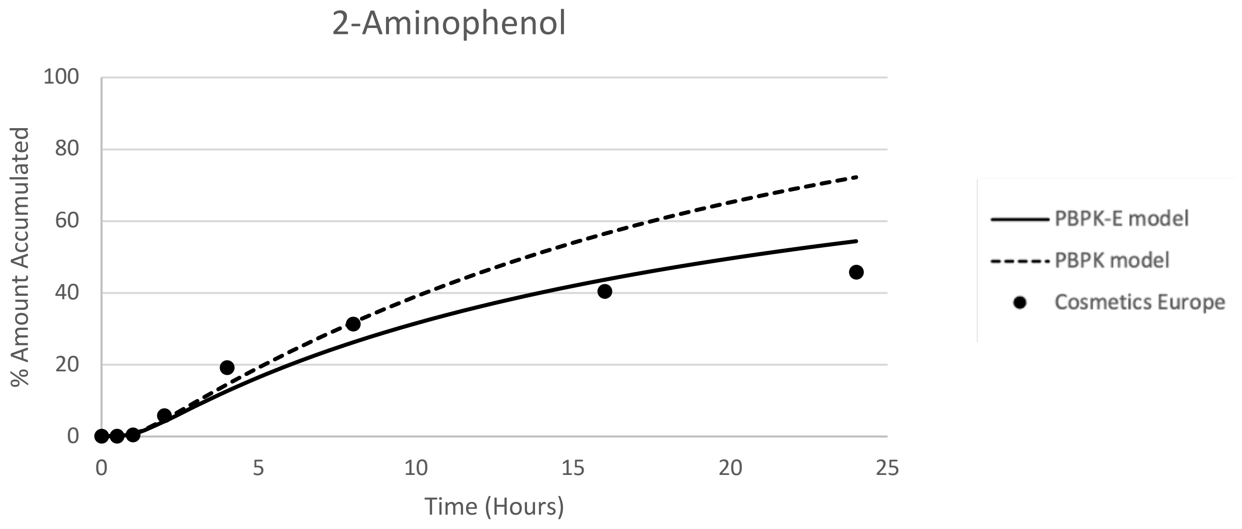

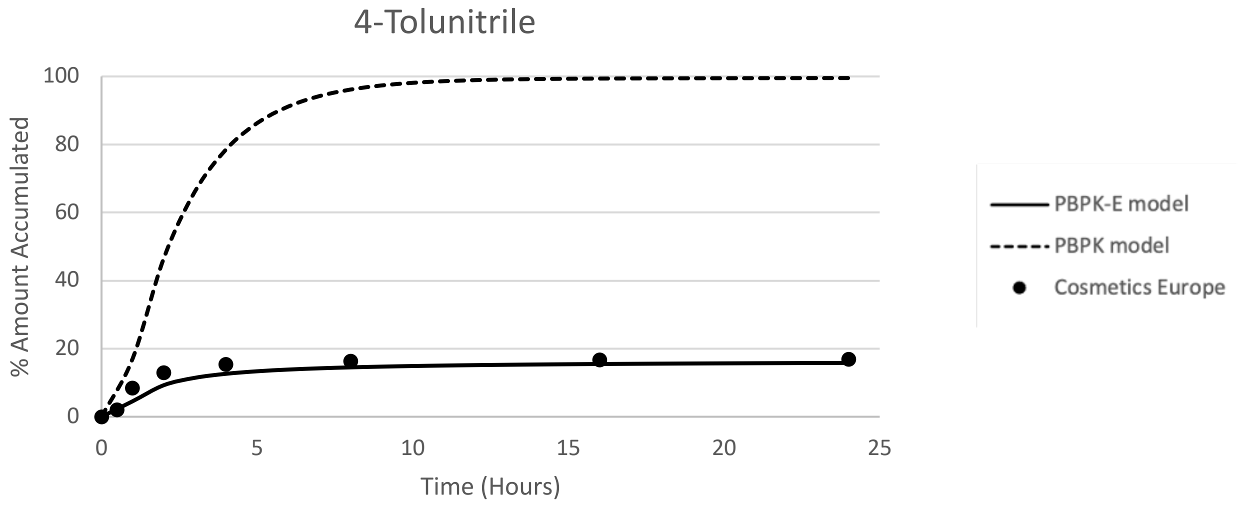

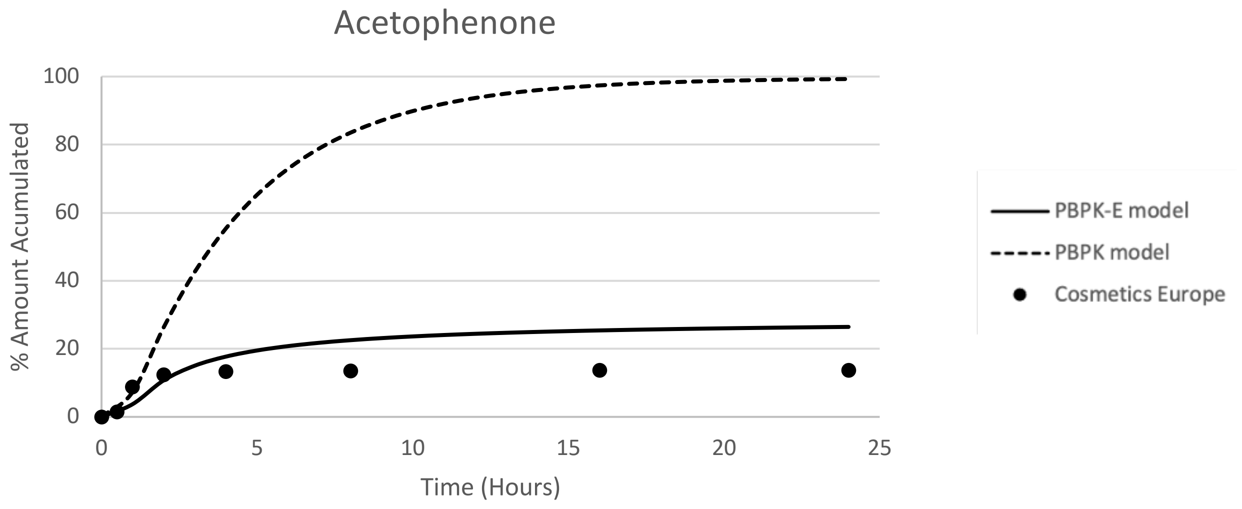

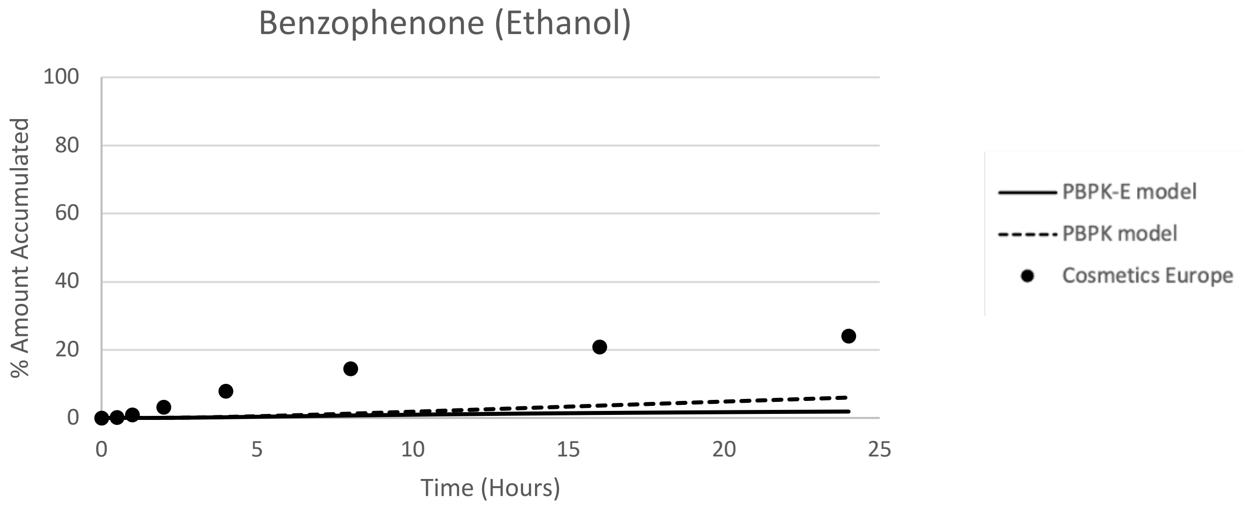
Benzophenone (Ethanol)
% Amount Accumulated
Time (Hours)
PBPK-E model
PBPK model
Cosmetics Europe

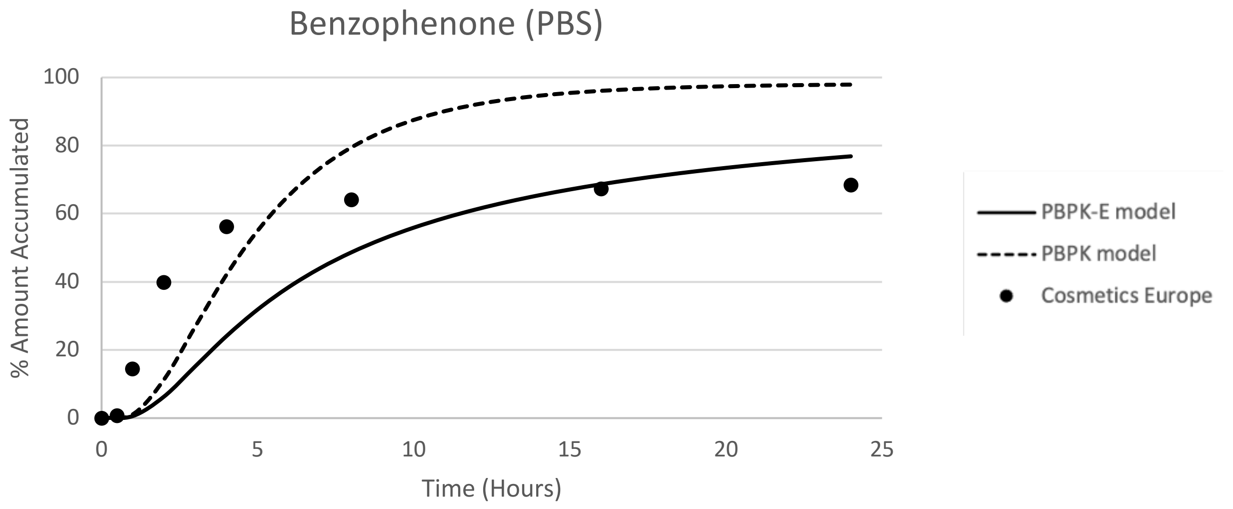
Benzophenone (PBS)
% Amount Accumulated
Time (Hours)
PBPK-E model
PBPK model
Cosmetics Europe

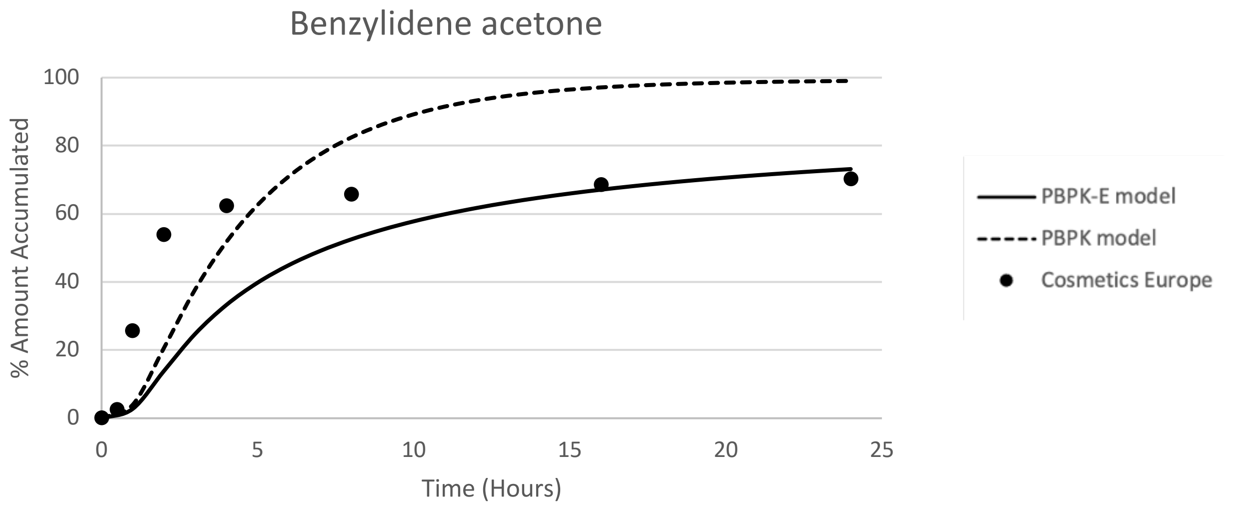
Benzylidene acetone
% Amount Accumulated
Time (Hours)
PBPK-E model
PBPK model
Cosmetics Europe

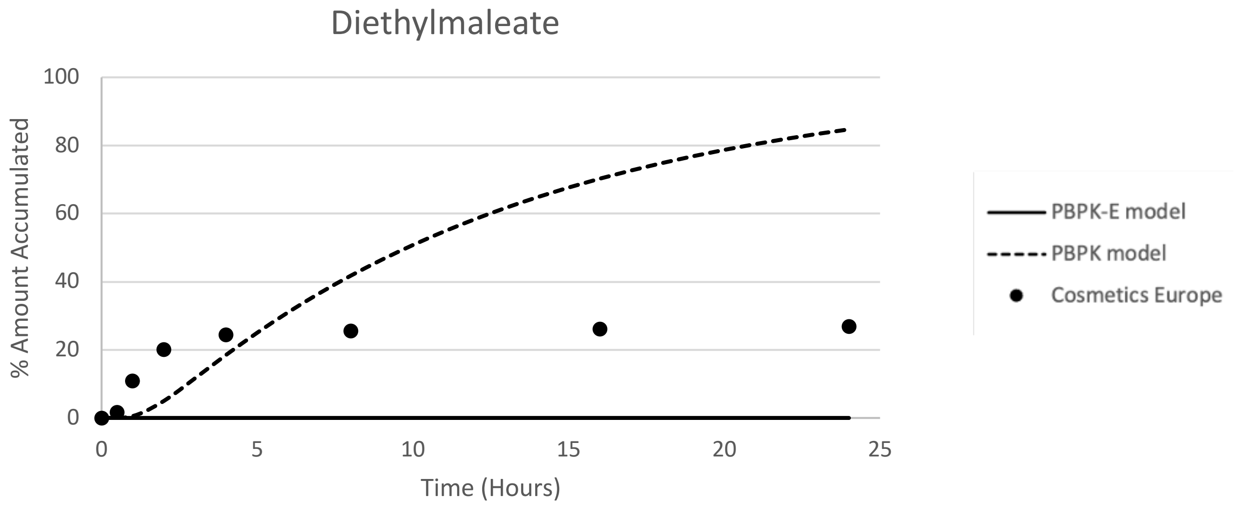
Diethylmaleate
100
80
60
40
20
0
% Amount Accumulated
0
5
10
15
20
25
Time (Hours)
PBPK-E model
PBPK model
Cosmetics Europe

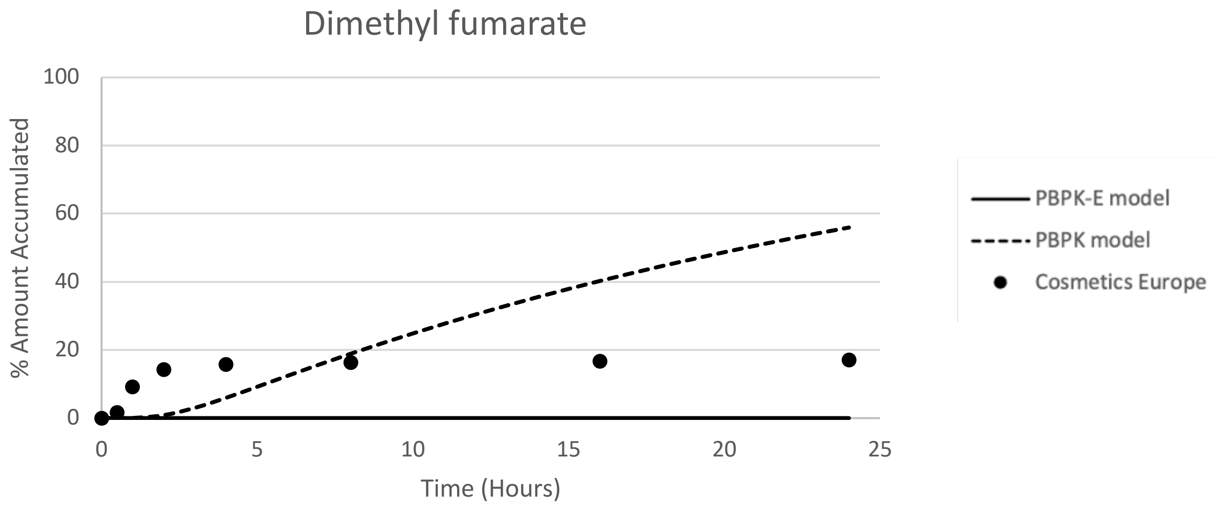
Dimethyl fumarate
100
80
60
40
20
0
% Amount Accumulated
0
5
10
15
20
25
Time (Hours)
PBPK-E model
PBPK model
Cosmetics Europe

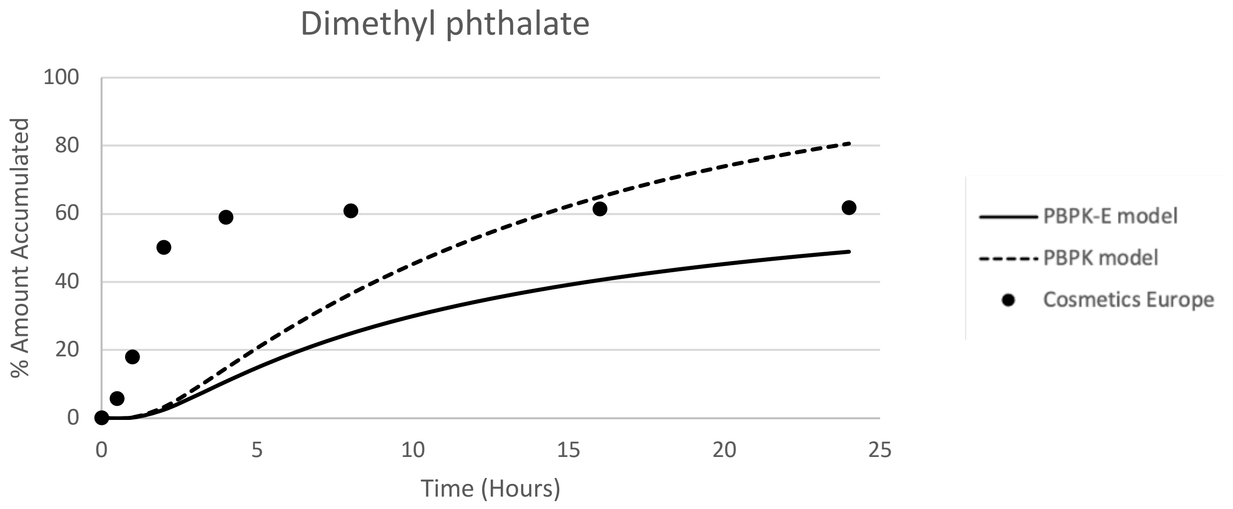
Dimethyl phthalate
100
80
60
40
20
0
% Amount Accumulated
0
5
10
15
20
25
Time (Hours)
PBPK-E model
PBPK model
Cosmetics Europe

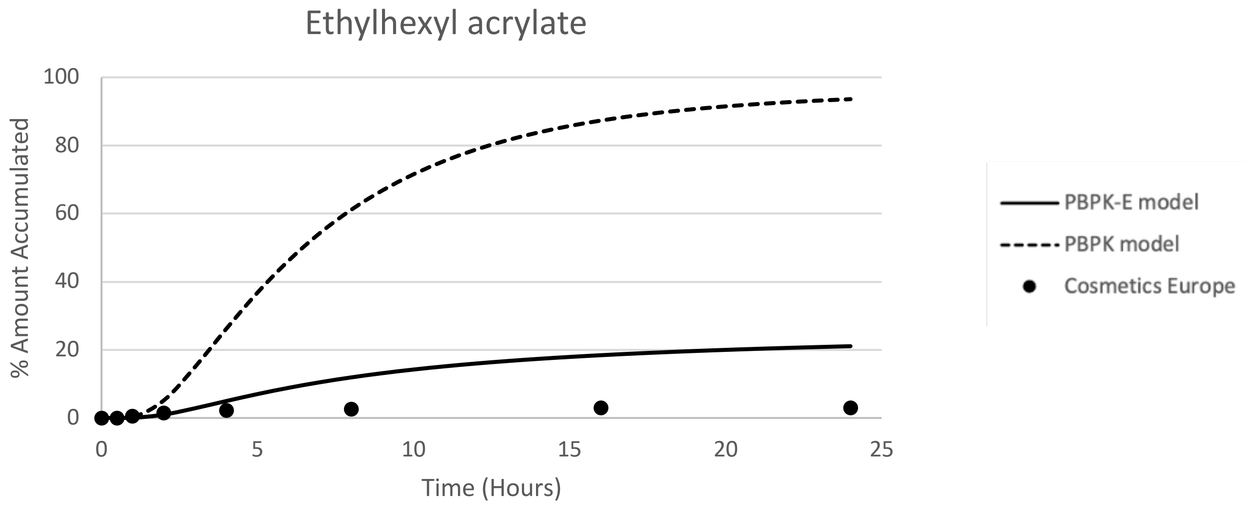

Ethylhexyl acrylate
% Amount Accumulated
Time (Hours)
PBPK-E model
PBPK model
Cosmetics Europe

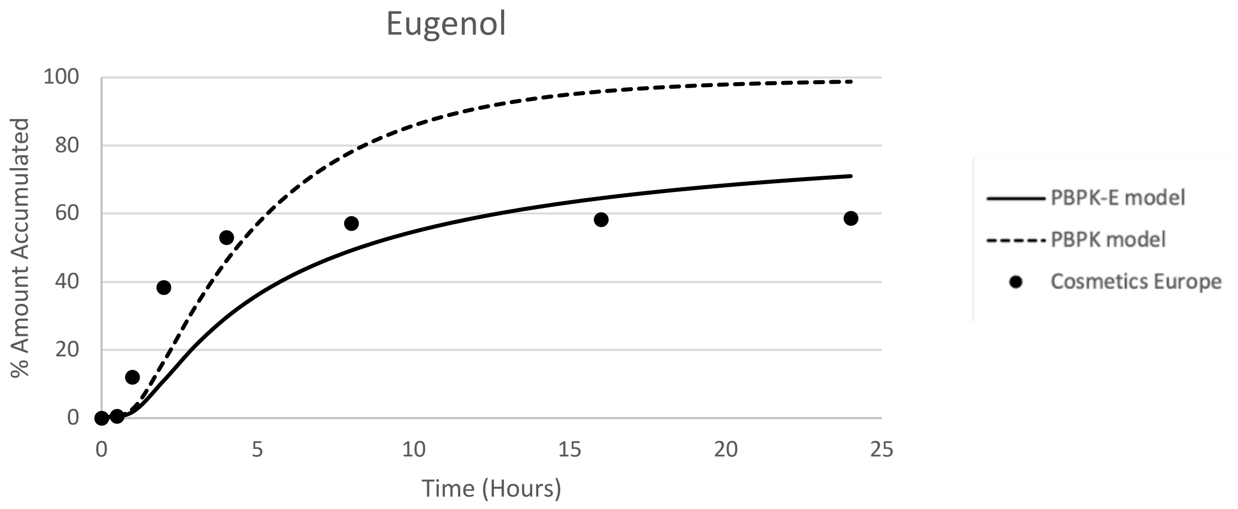

Eugenol
% Amount Accumulated
Time (Hours)
PBPK-E model
PBPK model
Cosmetics Europe

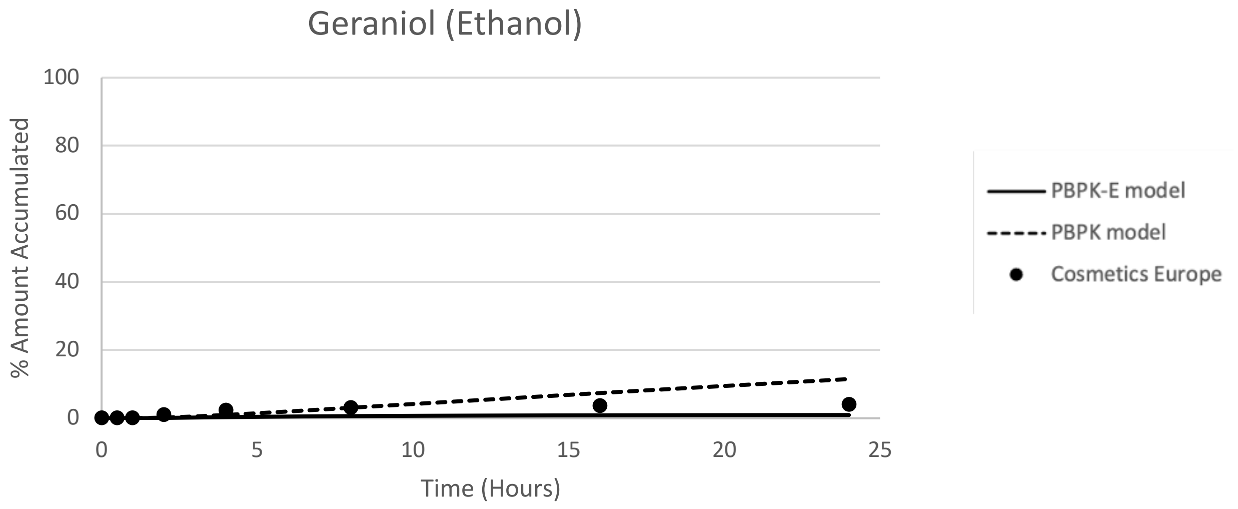

Geraniol (Ethanol)
% Amount Accumulated
Time (Hours)
PBPK-E model
PBPK model
Cosmetics Europe

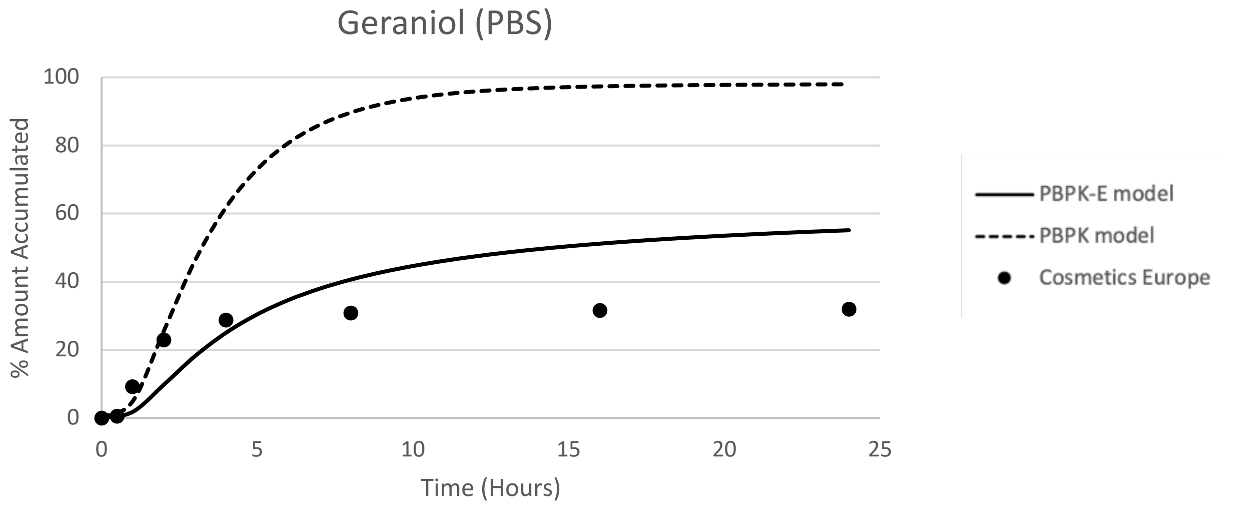
Geraniol (PBS)
% Amount Accumulated
Time (Hours)
100
80
60
40
20
0
0
5
10
15
20
25
PBPK-E model
PBPK model
Cosmetics Europe

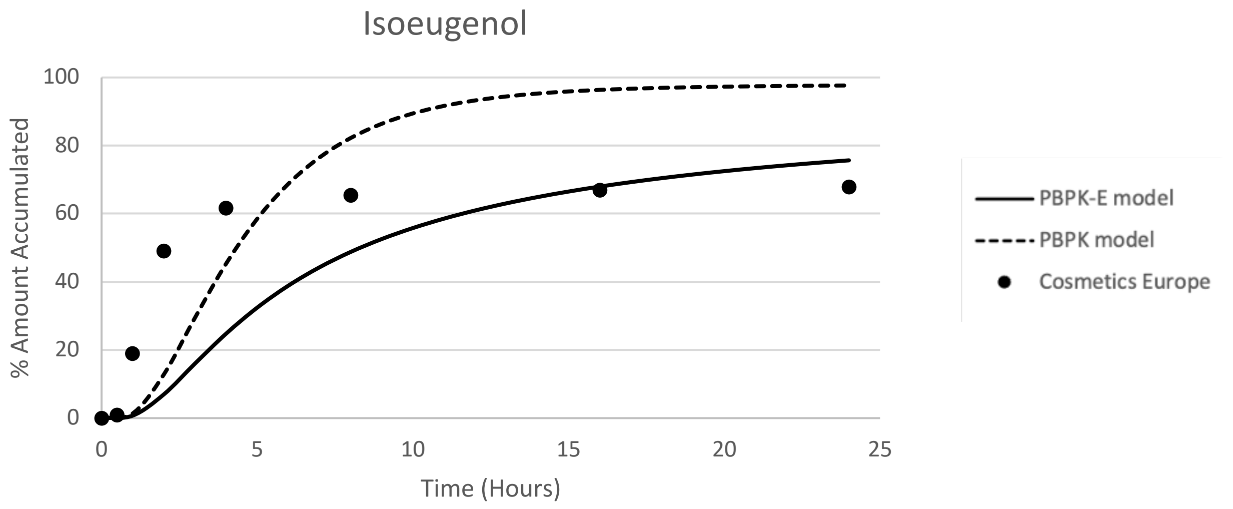
Isoeugenol
% Amount Accumulated
Time (Hours)
100
80
60
40
20
0
0
5
10
15
20
25
PBPK-E model
PBPK model
Cosmetics Europe

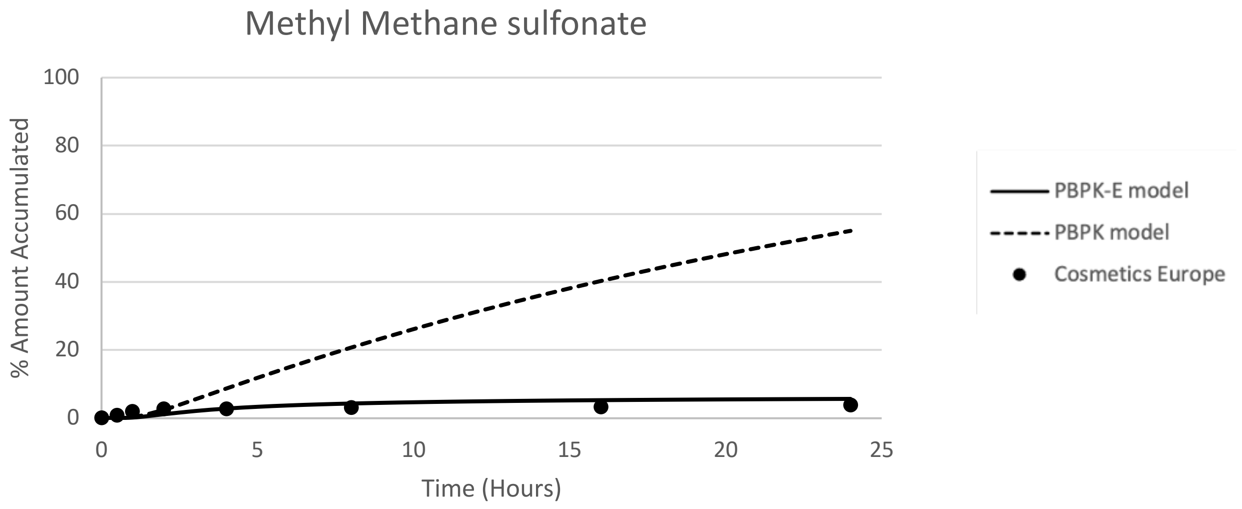
Methyl Methane sulfonate
% Amount Accumulated
Time (Hours)
100
80
60
40
20
0
0
5
10
15
20
25
PBPK-E model
PBPK model
Cosmetics Europe

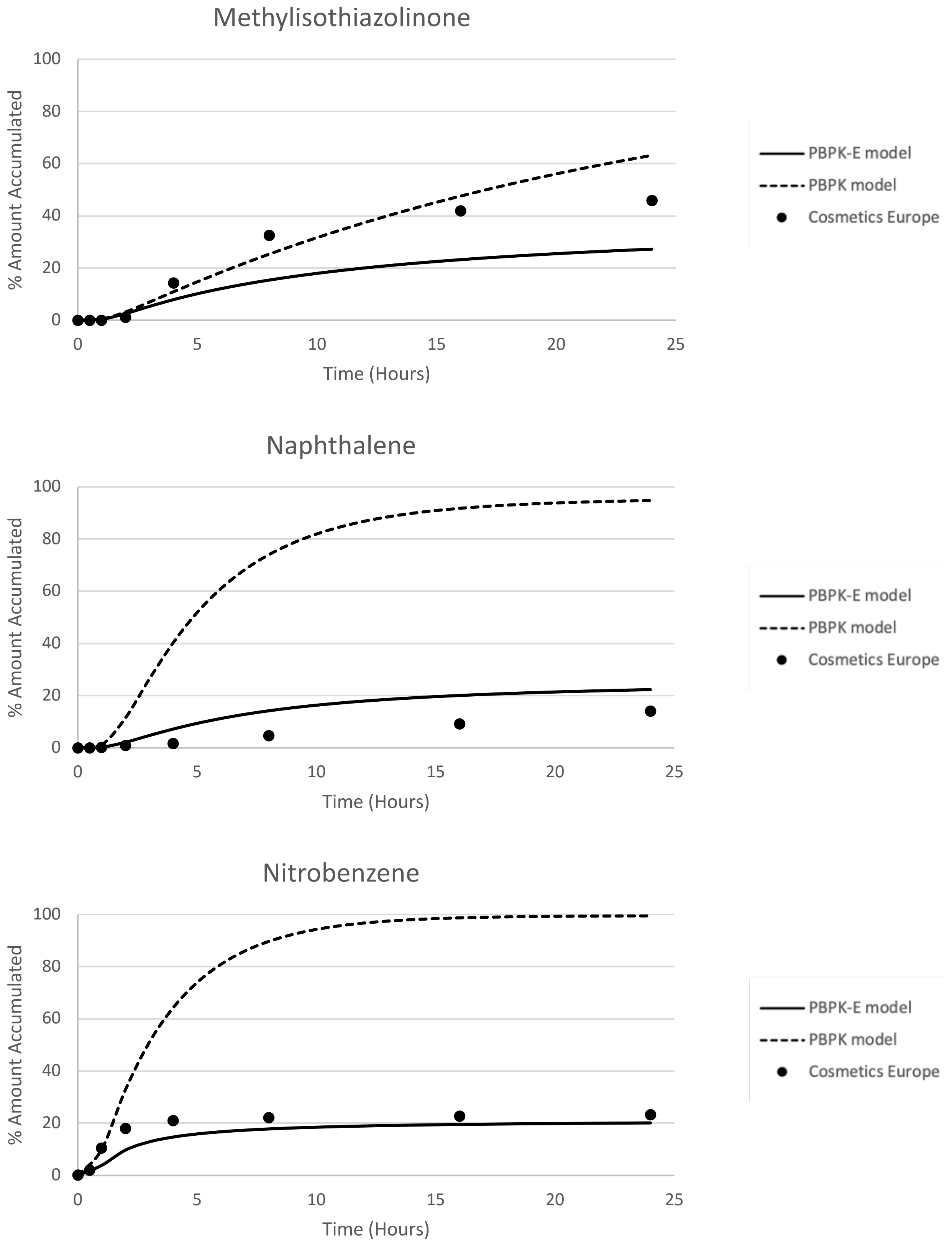
Methylisothiazolinone
% Amount Accumulated
Time (Hours)
PBPK-E model
PBPK model
Cosmetics Europe
Naphthalene
% Amount Accumulated
Time (Hours)
PBPK-E model
PBPK model
Cosmetics Europe
Nitrobenzene
% Amount Accumulated
Time (Hours)
PBPK-E model
PBPK model
Cosmetics Europe

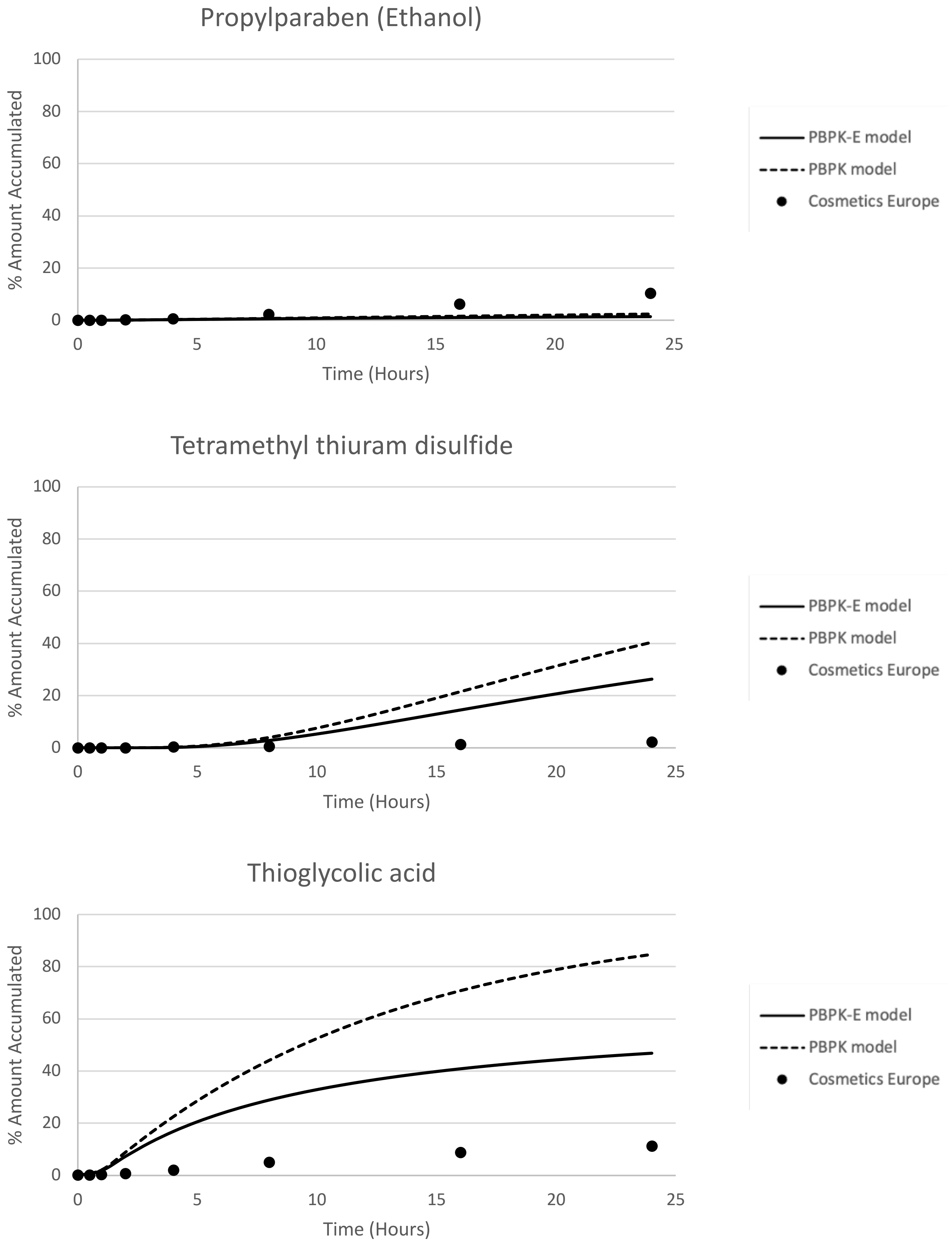
Propylparaben (Ethanol)
% Amount Accumulated
0
20
40
60
80
100
0
5
10
15
20
25
Time (Hours)
PBPK-E model
PBPK model
Cosmetics Europe
Tetramethyl thiuram disulfide
% Amount Accumulated
0
20
40
60
80
100
0
5
10
15
20
25
Time (Hours)
PBPK-E model
PBPK model
Cosmetics Europe
Thioglycolic acid
% Amount Accumulated
0
20
40
60
80
100
0
5
10
15
20
25
Time (Hours)
PBPK-E model
PBPK model
Cosmetics Europe

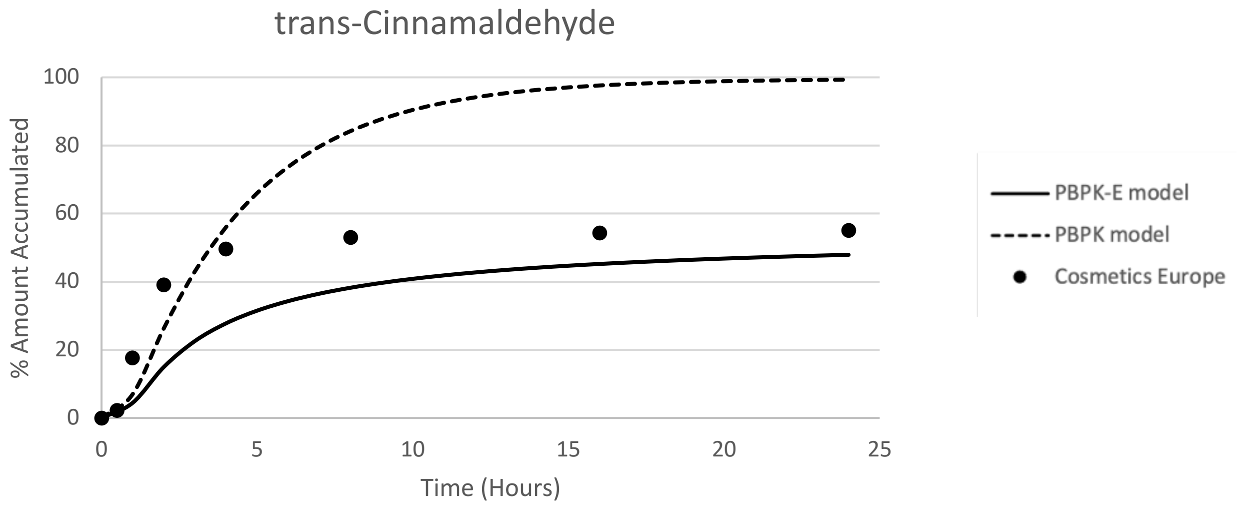
trans-Cinnamaldehyde
% Amount Accumulated
Time (Hours)
0
20
40
60
80
100
0
5
10
15
20
25
PBPK-E model
PBPK model
Cosmetics Europe

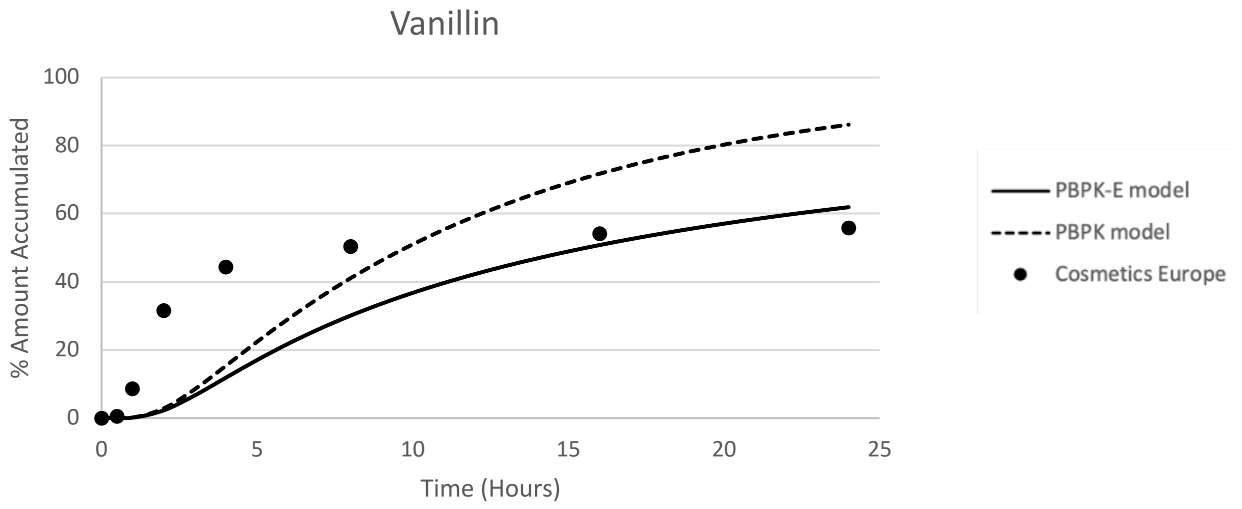
Vanillin
% Amount Accumulated
Time (Hours)
0
20
40
60
80
100
0
5
10
15
20
25
PBPK-E model
PBPK model
Cosmetics Europe
